# gfoRmula: An R Package for Estimating the Effects of Sustained Treatment Strategies via the Parametric g-formula

**DOI:** 10.1016/j.patter.2020.100008

**Published:** 2020-05-18

**Authors:** Sean McGrath, Victoria Lin, Zilu Zhang, Lucia C. Petito, Roger W. Logan, Miguel A. Hernán, Jessica G. Young

**Affiliations:** 1 Department of Biostatistics, Harvard T.H. Chan School of Public Health, Boston, MA 02115, USA; 2 School of Computer Science, Carnegie Mellon University, Pittsburgh, PA 15213, USA; 3 Department of Population Medicine, Harvard Medical School and Harvard Pilgrim Health Care Institute, Boston, MA 02215, USA; 4 Department of Medical Oncology, Dana Farber Cancer Institute, Boston, MA 02215, USA; 5 Feinberg School of Medicine, Northwestern University, Chicago, IL 60611, USA; 6 Department of Epidemiology, Harvard T.H. Chan School of Public Health, Boston, MA 02115, USA; 7 Harvard-MIT Division of Health Sciences and Technology, Cambridge, MA 02139, USA

**Keywords:** g-formula, longitudinal data, causal inference, R

## Abstract

Researchers are often interested in estimating the causal effects of sustained treatment strategies, i.e., of (hypothetical) interventions involving time-varying treatments. When using observational data, estimating those effects requires adjustment for confounding. However, conventional regression methods cannot appropriately adjust for confounding in the presence of treatment-confounder feedback. In contrast, estimators derived from Robins's g-formula may correctly adjust for confounding even if treatment-confounder feedback exists. The package **gfoRmula** implements in R one such estimator: the parametric g-formula. This estimator can be used to estimate the effects of binary or continuous time-varying treatments as well as contrasts defined by static or dynamic, deterministic, or random interventions, as well as interventions that depend on the natural value of treatment. The package accommodates survival outcomes as well as binary or continuous outcomes measured at the end of follow-up. This paper describes the **gfoRmula** package, along with motivating background, features, and examples.

## Highlights

• gfoRmula supports survival outcomes and binary/continuous end of follow-up outcomes
• gfoRmula has several options for handling competing events
• Users can specify custom covariate models, history functions, and interventions
• Users can incorporate deterministic knowledge and visit processes

## The Bigger Picture

Causal inference is a core task of data science. When data from randomized experiments are not available, data analysts often rely on nonexperimental (observational) data to estimate causal effects. The parametric g-formula is a statistical method to estimate the causal effects of sustained treatment strategies from observational data with time-varying treatments, confounders, and outcomes. Although this methodology was introduced in the 1980s, it has not been widely used due to the lack of open-source software. This article presents the gfoRmula package, an implementation of the parametric g-formula in R. The aim of this software is to facilitate the application of the parametric g-formula to complex, observational data to answer causal questions. Furthermore, this package helps provide a way to compare the performance of the parametric g-formula to other methods in the causal inference literature.

## Teaser

McGrath et al. present the statistical software package, gfoRmula. This package implements the parametric g-formula, a statistical method to estimate the causal effects of sustained treatment strategies from observational data with time-varying treatments, confounders, and outcomes.

## Introduction

Researchers are often interested in using longitudinal data to estimate the causal effects of hypothetical interventions that are sustained over time (equivalently, “treatment strategies,” “rules,” and “policies”) on a population outcome mean. Standard regression methods for confounding control generally fail to recover such causal effects, which involve time-varying treatments, when time-varying confounders are themselves affected by past treatment.^1^ For example, in studies of the effect of time-varying antiretroviral treatment strategies on long-term mortality risk in HIV-positive individuals, CD4 cell count is a time-varying confounder, i.e., at follow-up time *k* it is a risk factor for both future treatment and mortality. It is also, itself, affected by past treatment.

In such settings, alternative estimators derived from Robins's “g-formula” may recover effects of sustained treatment interventions. The g-formula characterized by a user-specified treatment strategy is a function of the observed longitudinal data. Under untestable assumptions, including that sufficient covariates are measured to adjust for confounding,^2^ this data function equals the outcome mean/risk had that intervention been implemented in all individuals in the study population. The package **gfoRmula** (current version 0.3.1) implements in R one estimator of the g-formula: “the parametric g-formula,”^3^^,^^4^ available at https://CRAN.R-project.org/package=gfoRmula. In general, the g-formula is a high-dimensional sum or integral over a function of (1) the observed outcome mean (or, in survival settings, time-varying hazards) conditional on each observed treatment and confounder history, (2) the observed joint distribution of the confounders at each time *k* conditional on each observed treatment and confounder history, and (3) an intervention density defining the time-varying user-specified treatment strategy. The parametric g-formula estimates (1) and (2) via parametric models and approximates the sum via a Monte Carlo simulation.

The **gfoRmula** package accommodates general interventions or treatment strategies, which may be “static” or “dynamic”^4^, ^5^, ^6^, ^7^, ^8^, ^9^, ^10^ and, further, “deterministic” or “random.”^11^, ^12^, ^13^, ^14^, ^15^ It also may be used to estimate effects of treatment strategies that depend on the “natural value of treatment” at *k*; the value of treatment that would have been observed at *k* were the intervention discontinued directly before *k*.^16^ The package also allows: (1) binary or continuous/multilevel time-varying treatments; (2) different types of outcomes (survival or continuous/binary end of follow-up); (3) data with competing events (survival outcomes) and loss to follow-up; (4) joint interventions on multiple treatments and other options.

The **gfoRmula** R package has many of the capabilities of the **GFORMULA** SAS macro,^17^ as well some overlapping capabilities described for the **gformula** command in Stata,^18^ to implement the parametric g-formula. However, the **gfoRmula** R package will facilitate use of this approach for many researchers who may only be able (or prefer) to conduct data analysis in R given its free and open-source (non-proprietary) platform. The **gfoRmula** R package also more easily allows users to incorporate their own helper functions in different steps of the algorithm.

In this article, we describe input dataset requirements and core features of the package along with various code examples. Theoretical background including formal definition of the observed data structure, causal estimands, and identifying assumptions, along with details of the estimation algorithm and some advanced package features, are provided in appendices.

## Results

In this section, we describe how to use the **gfoRmula** package and provide complete coding examples.

### Using the **gfoRmula** Package

#### Specifying the Outcome Type

The main function in the **gfoRmula** package is the gformula function. The argument outcome_type specifies the type of the outcome of interest; 'survival' should be used for survival outcomes (e.g., an indicator of all-cause mortality by an end of follow-up time K+1); 'continuous_eof' should be used for fixed continuous end of follow-up outcomes (e.g., blood pressure at time K+1); and 'binary_eof' should be used for fixed binary end of follow-up outcomes (e.g., indicator of high cognitive score at K+1). For survival outcomes, the package outputs estimates of contrasts in failure risks by a specified follow-up time k≤K+1 (i.e., the probability of failing by that time) under different user-specified treatment strategies. For end of follow-up outcomes, it outputs estimates of contrasts in the outcome mean at K+1.

#### Required Structure of the Input Dataset

The input dataset to the gformula function must be an R data.table. For all outcome types, the input dataset should contain one record for each follow-up time *k* for each subject present at baseline (specified by the parameter id). The time index *k* must start at 0 (indexing baseline) and increase in increments of 1. Each column of the dataset will index one of *p* time-varying covariates $Z_{j,k}$, j=1,…,p. Additional columns will contain values of time-fixed baseline confounders (e.g., race, sex) with values repeated on each line *k* for that subject in the dataset.

In some cases, additional records (rows) may be specified containing pre-baseline information. If no records before baseline are needed to control for confounding, the parameter time_name is an index that specifies the follow-up time *k*, i.e., the value of time_name for each subject starts at 0 and increases in increments of 1 until, at most, *K*. If records before baseline are needed, time_name can specify the index of the pre-baseline time and the follow-up time *k*. Letting *i* denote the maximum number of pre-baseline times contained in the data, the value of time_name for each subject starts at −i and increases in increments of 1 until, at most, *K*. Each subject can then have a maximum of K+1+i records with K+1, the end of follow-up of interest and i=0 when no additional pre-baseline records are included. See Appendix C.1 for additional coding specifications in the case of i≠0. In the remainder of the text, we presume i=0 unless otherwise specified.

A subject who is censored in interval k+1 $\left(C_{k}=0,C_{k}+1=1\right)$ will have k+1 records with time index *k* on the last line. Other requirements are dependent on the outcome type, which we describe below.

##### Survival Outcomes

In this case, the dataset should additionally contain a column $Y_{k+1}$ indicating on line *k* whether the event of interest occurred by the next interval. A subject who first has the event of interest in interval k+1 will have only k+1 follow-up records with time index *k* on the last line. If $C_{k+1}=1$ then the user has the choice to code $Y_{k+1}$ as either NA or 0. The user does not need to include the column with a time-varying censoring indicator in the dataset. For additional notes on construction of the input dataset when there is censoring, including the choice of how to code $Y_{k+1}$ and why a censoring indicator column is not needed, see the end of Appendix B.1.

If the event of interest is subject to a competing event for some subjects and some *k* and the user chooses to treat competing events as censoring events, competing events should be treated like any component of $C_{k+1}$ and coding requirements above must be applied. In turn, in this case, the input dataset does not require a variable with competing event status. If, alternatively, competing events are not treated as censoring events, the user must include in the input dataset a column with a time-varying indicator of the competing event $D_{k+1}$. If $D_{k+1}=1$ on a line *k* for a given subject, that subject will only have k+1 lines in the follow-up data with follow-up time *k* on the last line and, on that line, $Y_{k+1}$ must be coded NA. See the datasets basicdata_nocomp and basicdata included with the package for examples of input datasets for survival outcomes without and with competing events, respectively.

When competing events are treated as censoring events, the algorithm targets a particular direct effect, i.e., a contrast of failure risks under different treatment strategies and an additional intervention that (somehow) eliminates competing events. Otherwise, the algorithm targets a particular total effect, i.e., this contrast but without elimination of competing events. The total effect is precisely a contrast of different (counterfactual) cause-specific cumulative incidences as defined in the classical survival analysis literature.^19^^,^^20^ For details see Young et al.^21^ and Appendix A.

The user can specify the parameter time_points to be a follow-up interval k<K+1 (with K+1 the maximum number of records for an individual in the dataset) in order to compute the risk estimates through k<K+1. The default value of time_points is K+1.

##### End of Follow-Up Outcomes

In this case, the dataset should additionally contain a column *Y* with the value of the outcome in interval K+1. Only the value of this variable on line k=K is used in the algorithm. Death, if present, must be treated as a censoring event. For subjects who are first censored in interval K+1, these subjects will have K+1 follow-up records in the data, and *Y* on line k=K must be coded as NA. For subjects censored prior to interval K+1, the value of *Y* will be ignored in the estimation algorithm. See the dataset continuous_eofdata included with the package for an example of an input dataset for an end of follow-up outcome.

#### Specifying the Input Dataset, Individual Identifier, Time Index, Outcome, and Competing Events

The input dataset is specified by the argument obs_data. The column of this dataset containing the individual identifier is specified by the argument id, the time index by the argument time_name, the outcome by the argument outcome_name, and, if present and not defined as a censoring event, competing event status by the argument compevent_name. Sample syntax for a survival outcome is given by:

gformula(…, outcome_type = 'survival',

 obs_data = obs_data,

 id = 'ID',

 time_name = 't0',

 outcome_name = 'Y',

 compevent_name = 'D')

#### Specifying Time-Varying and Baseline Covariates

The time-varying covariates $Z_{k}$ are defined by the vector argument covnames. Time-fixed baseline confounders are defined by the vector argument basecovs. Sample syntax is given by:

gformula(…, covnames = c('Z1', 'Z2', 'Z3'),

 basecovs = c('race','sex'))

#### Generating Covariate Histories

As detailed in Appendix B, the parametric g-formula estimates the g-formula indexed by a user-specified treatment strategy in two steps. In step 1, (i) the observed outcome mean (or, in survival settings, time-varying hazards) conditional on each observed treatment and confounder history and (ii) the observed joint distribution of the confounders at each follow-up time *k* conditional on each observed treatment and confounder history are estimated. The joint distribution is estimated as a product of conditional distributions based on an arbitrarily ordered factorization of the *p* components of $Z_{k}$ (see Appendix B for additional details and example below). In step 2, a Monte Carlo simulation is conducted in which the covariates are iteratively simulated at each follow-up time *k* using these estimates, where the simulated value of treatment is replaced with the value consistent with the user-specified treatment strategy.

The arguments histvars and histories must be used to specify any desired functions of history that will be used for estimation in step 1 (and then, in turn, in the simulation in step 2) of the estimation algorithm that cannot be defined within a model statement in R. For example, consider a time-varying covariate $Z_{4,k}$ named Z4 in the input dataset and suppose the user assumes that the distributions of “future” covariates ordered under this arbitrary factorization of the vector $Z_{k}$, $Z_{j,k}$, j>4, depend on the history of $Z_{4,k}$ through the cumulative average of this variable through each *k*; i.e., $\frac{1}{k}\sum_{t=0}^{k}Z_{4,t}$ for k>0 and $Z_{4,0}$ otherwise. The user must then use histvars and histories to create in the dataset obs_data this additional time-varying covariate containing the cumulative average of $Z_{4,k}$ at each *k*.

Pre-coded functions of history for a covariate named Zj in the input dataset include:

• lagged: this adds a variable to the specified input dataset named lagi_Zj, containing the *i*th lag of Zj relative to the current follow-up time *k* (i.e., its value at k−i for k≥i) for all i=1,…,r with *r* the desired number of lags. lagi_Zj is set to 0 on lines with k<i.
• cumavg: this adds a variable to the specified input dataset named cumavg_Zj, which contains the cumulative average of Zj up until the current follow-up time k>0. It is set to Zj at k=0.
• lagavg: this adds a variable to the specified input dataset named lag_cumavgi_Zj which contains the *i*th lag of the cumulative average of Zj relative to the current follow-up time *k*, i=1,…,r. lag_cumavgi_Zj is set to 0 on lines with k<i.

Note the desired number of lags *r* is specified using the argument covmodels (see the next section, Specifying Covariate Distributions).

The package will apply the function of history listed in the *q*th element of the vector argument histories to all variables listed in the *q*th element of the list argument histvars. Therefore, the length of histvars and histories need to match. Sample syntax that would add lagged, cumulative average, and lagged cumulative average functions of the covariates Z1, Z2, and Z3 as new variables to the input dataset obs_data for use in estimation of conditional distributions/hazards/means (see the sections Specifying Covariate Distributions and Specifying Outcome and Competing Event Models) is shown in Box 1.

Box 1 gformula(…, covnames = c('Z1', 'Z2', 'Z3’),

 histories = c(lagged, cumavg, lagavg),

 histvars = list(c('Z1', 'Z2', 'Z3’), c('Z1', 'Z2', 'Z3’),

  c('Z1', 'Z2', 'Z3’)))

Throughout this subsection, it was assumed that data on time-varying covariates at pre-baseline are either not available or not necessary to adjust for confounding. See Appendix C.1 for how these history functions are applied when additional records containing pre-baseline values of time-varying covariates are included in the input dataset.

#### Specifying Covariate Distributions

The vector argument covtypes is used to specify the distributions of each time-varying covariate $Z_{j,k}$ conditional on a function of history $\left(Z_{j-1,k},\ldots ,Z_{1,k},{\bar{Z}}_{k-1}\right)$, j=1,…,p, and k=0,…,K, how parameters of those distributions will be estimated, and, in turn, how covariate values in step 2 of the algorithm will be simulated. The **gfoRmula** package supplies a number of pre-programmed options for input to the argument covtypes, which are described in detail in Appendix C.2: 'binary', 'normal', 'categorical', 'bounded normal', 'zero-inflated normal', 'truncated normal', 'absorbing', and 'categorical time'. Each of the elements of covtypes requires its own set of specific sub-parameters, which are contained within the list argument covparams. Each sub-parameter vector within this list must be the same length as covnames and covtypes. Several examples are also given later in the Examples section.

#### Specifying Outcome and Competing Event Models

The argument ymodel takes as input an R model statement. For survival outcomes, this model statement is passed to glm in R with binomial family and logit link to estimate the hazard at each follow-up time *k* conditional on history (i.e., $p_{k}\left({\bar{l}}_{k},{\bar{a}}_{k}\right)$ from step 1.b of the algorithm in Appendix B.1). Therefore, this model should generally depend on a function of *k*.

For continuous end of follow-up outcomes, this model statement is passed to glm with gaussian family and identity link to estimate the mean of the outcome at end of follow-up conditional on time-varying and baseline covariate history (i.e., $\mu \left({\bar{l}}_{K},{\bar{a}}_{K}\right)$ from modified step 2.c of the algorithm in Appendix B.2). Therefore, this model is not dependent on follow-up time *k*. Similarly, for binary end of follow-up outcomes, this model statement is passed to glm with binomial family and logit link to estimate the mean of the outcome at end of follow-up conditional on history (which is not dependent on *k*).

The argument compevent_model takes as input an R model statement passed to glm in R with binomial family and logit link to estimate the (cause-specific) hazard of the competing event at each follow-up time *k* conditional on history (i.e., $q_{k}\left({\bar{l}}_{k},{\bar{a}}_{k}\right)$ from step 1.c of the algorithm in Appendix B.1). The model is generally dependent on *k*. This argument should only be specified for survival outcomes and when the user chooses not to define the competing event as a censoring event. Several syntax examples are given in Examples.

#### Specifying the Interventions

We define an “intervention rule” as a strategy involving an intervention on one or more treatments. However, we will use the terms ”intervention,” “intervention rule,” and “strategy” interchangeably with “joint interventions” referring to strategies with interventions on more than one treatment.

The arguments intvars, interventions, and int_times are jointly used to define the user-specified treatment interventions to be compared. See Appendix A.2 for a review of classification of interventions (static versus dynamic, deterministic versus random).

intvars is a list of vectors. The number of vector elements of this list should be the number of user-specified interventions of interest. Each vector element specifies the time-varying covariates to be intervened upon under that intervention (i.e., the treatments/exposures). This vector will have a single element if the intervention involves intervening on only a single time-varying covariate (we will call this a “single intervention”) and will have multiple elements if the intervention involves intervening on multiple time-varying covariates (a “joint intervention”).

interventions is a list whose elements are lists of vectors. We refer to the list specified for interventions as the “outer list” and refer to the elements of this outer list as the “inner lists”. The length of the outer list should be the number of user-specified interventions of interest. Each inner list specifies one or more treatment strategies, each one defined by a vector of arguments. The inner list will include only a single vector for an element corresponding to a single treatment strategy and will include multiple vectors for an element corresponding to a joint intervention (each vector element specifying the intervention for a given covariate under that intervention). The first element of a vector specifying an intervention for a given covariate must be the function that defines the intervention, and the following elements specify the input parameter(s) of the intervention function (if applicable). Box 2 includes a list of pre-coded interventions or treatment strategies available with the package with examples (see Examples for additional examples).

Box 2

• static: the function static specifies a deterministic static intervention. It requires specification of a vector of length time_points with the *k*th element the value of treatment to be assigned under that intervention at follow-up time *k*. The following is sample syntax to compare two static interventions on a binary time-varying covariate A that sets this variable to 0 at all follow-up times versus 1 at all follow-up times:

gformula(…, time_points = 10,

 intvars = list(c('A'), c('A')),

 interventions = list(list(c(static, rep(0, 10))),

  list(c(static, rep(1, 10)))))

The following extends this example to compare joint static interventions that sets two time-varying covariates A1 and A2 to 0 at all follow-up times versus 1 at all follow-up times:

gformula(…, time_points = 10,

 intvars = list(c('A1', 'A2'), c('A1', 'A2')),

 interventions = list(list(c(static, rep(0, 10)),

 c(static, rep(0, 10))),

 list(c(static, rep(1, 10)),

 c(static, rep(1, 10)))))

• threshold: the function threshold specifies a threshold intervention, an example of an intervention at *k* that depends on the natural value of treatment *k* (see Appendix A.2). Under a threshold intervention, at each follow-up time *k*, the treatment value is set to the simulated (natural) value of treatment at *k* if it falls within a user-specified range and, otherwise, the treatment value under intervention is set to the maximum of that range (if the simulated value is above) or the minimum of that range (if the simulated value is below).^13^^,^^22^^,^^23^ This treatment strategy requires specification of the minimum and maximum of the desired range. If no maximum is desired this should be set to Inf and if no minimum is desired this should be set to -Inf. The following is sample syntax to compare two threshold interventions maintaining a time-varying covariate Z2 at all follow-up times at ≥2 versus ≥3:

gformula(…, intvars = list(c('Z2'), c('Z2')),

 interventions = list(list(c(threshold, 2, Inf)),

 list(c(threshold, 3, Inf))))

• natural: the function natural specifies the natural course intervention under which the simulated value is assigned at each follow-up time *k*. Risks/means under the natural course are always computed by default; thus, this intervention does not need to be specified.

To specify the intervention used as the “reference,” i.e., the denominator in the risk/mean ratio/difference calculations, set ref_int equal to the index of interventions in which the desired reference intervention is specified. By default, ref_int = 0, or the natural course.

Optionally, users can specify the time points in which interventions are applied via the int_times argument. As with the intvars argument, int_times is a list whose elements are lists of vectors. Each vector specifies the time points at which the relevant intervention is applied on the corresponding variable in intvars. When an intervention is not applied at a follow-up time *k* (not included in int_times), the simulated (natural) value is used. By default, all interventions are applied at all follow-up time points. In Box 3, the extension of the above example compares two interventions on the time-varying covariate Z2 under which the natural value of treatment is assigned at k=0,1 and, for k≥2, a threshold intervention is applied that ensures the treatment is assigned values ≥2 versus ≥3.

Box 3 gformula(…, time_points=10,

 intvars = list(c('Z2'), c('Z2')),

 interventions = list(list(c(threshold, 2, Inf)),

 list(c(threshold, 3, Inf))),

 int_times = list(list(2:9), list(2:9)))

#### Output

The gformula function returns an S3 class object (of class gformula), which is a list that contains the following main elements: (1) a data.table containing the nonparametric estimates of the natural course risk/mean outcome (see Appendix B) and the parametric g-formula estimates of the risk/mean outcome under each specified intervention at each follow-up time point; (2) the coefficients, standard errors, variance-covariance matrices of the parameters of the fitted models, and RMSE values of the models fit in step 1 of the algorithm; and optionally, (3) the fitted models (e.g., glm objects) in step 1 of the algorithm and (4) the simulated histories from step 2 of the algorithm under each specified intervention. Objects returned by the gformula function also have class gformula_survival (for survival outcomes), gformula_continuous_eof (for continuous end of follow-up outcomes), or gformula_binary_eof (for binary end of follow-up outcomes).

S3 print, summary, plot, coef, and vcov methods are available for objects returned by the gformula function. The plot method plots parametric g-formula versus nonparametric estimates of the risks by each follow-up interval under the natural course intervention for gformula_survival-type objects, along with parametric versus nonparametric estimates of covariate means under the natural course for gformula_survival, gformula_continuous_eof, and gformula_binary_eof-type objects. See also Young et al.^10^

### Examples

In this subsection, we provide complete examples along with a description of output.

#### Example 1: Estimating the Effect of Static Treatment Strategies on Risk of a Failure Event

The example dataset basicdata_nocomp consists of 13,170 observations on 2,500 individuals with a maximum of 7 follow-up times. No individuals are censored in this dataset. The variables in the dataset are:

t0: The time index

id: A unique identifier for each individual

L1: A time-varying covariate; binary

L2: A time-varying covariate; continuous

L3: A baseline covariate; continuous

A: The treatment variable; binary

Y: The outcome/failure event of interest; time-varying indicator of failure

Data for the first subject, who survives the whole follow-up, is presented below:

**Table:** 

	id	t0	L1	L2	L3	A	Y
1:	1	0	0	1.1470920	5	1	0
2:	1	1	0	−0.9254032	5	1	0
3:	1	2	0	−0.9899824	5	0	0
4:	1	3	1	1.0057421	5	1	0
5:	1	4	1	−1.1956468	5	1	0
6:	1	5	0	−0.9697723	5	1	0
7:	1	6	1	−1.0887002	5	1	0

The subject identifier id does not have to be a number as long as it is unique for each individual.

The syntax in Box 4 can be used to estimate, using this dataset, the risk of failure from the event of interest by K+1=7 under a strategy that sets treatment to 0 at all follow-up times for all individuals (“never treat”) and a strategy that sets it to 1 at all times (“always treat”). Note that we must use the nsimul parameter to set the number of simulated histories (*s*, Appendix B) because in this example the baseline sample size is n<10,000.

Box 4 > outcome_type <- 'survival'

> id <- 'id'

> time_points <- 7

> time_name <- 't0'

> covnames <- c('L1', 'L2', 'A')

> outcome_name <- 'Y'

> covtypes <- c('binary', 'bounded normal', 'binary')

> histories <- c(lagged, lagavg)

> histvars <- list(c('A', 'L1', 'L2'), c('L1', 'L2'))

> covparams <- list(covmodels = c(L1 ∼ lag1_A + lag_cumavg1_L1 + lag_cumavg1_L2 +

+  L3 + t0,

+  L2 ∼ lag1_A + L1 + lag_cumavg1_L1 +

+  lag_cumavg1_L2 + L3 + t0,

+  A ∼ lag1_A + L1 + L2 + lag_cumavg1_L1 +

+  lag_cumavg1_L2 + L3 + t0))

> ymodel <- Y ∼ A + L1 + L2 + L3 + lag1_A + lag1_L1 + lag1_L2 + t0

> intvars <- list('A', 'A')

> interventions <- list(list(c(static, rep(0, time_points))),

+  list(c(static, rep(1, time_points))))

> int_descript <- c('Never treat', 'Always treat')

> nsimul <- 10000

> gform_basic <- gformula(obs_data = basicdata_nocomp,

+  outcome_type = outcome_type, id = id,

+  time_points = time_points, time_name = time_name,

+  covnames = covnames, outcome_name = outcome_name,

+  covtypes = covtypes, covparams = covparams,

+  ymodel = ymodel, intvars = intvars,

+  interventions = interventions,

+  int_descript = int_descript, histories = histories,

+  histvars = histvars, basecovs = c('L3’),

+  nsimul = nsimul, seed = 1234)

> gform_basic

PREDICTED RISK UNDER MULTIPLE INTERVENTIONS

Intervention  Description

0  Natural course

1  Never treat

2  Always treat

Sample size = 2500, Monte Carlo sample size = 10000

Number of bootstrap samples = 0

Reference intervention = natural course (0)

k  Interv.  NP risk  g-form risk  Risk ratio  Risk difference

6  0  0.5056  0.5048280  1.0000000  0.0000000

6  1  NA  0.7314631  1.4489355  0.2266352

6  2  NA  0.2339747  0.4634741  −0.2708533

In this example, the parametric g-formula estimates of the risk (g-form risk) by end of follow-up under “never treat,” “always treat,” and the natural course is 0.731, 0.234, and 0.505, respectively. The risk ratio by K+1 comparing “never treat” with the natural course is 1.45. The nonparametric estimate (NP risk) of the natural course risk is 0.506 and close to the parametric g-formula estimate of this risk. This supports (but does not guarantee) the absence of gross model misspecification.^10^

**Figure 1:**
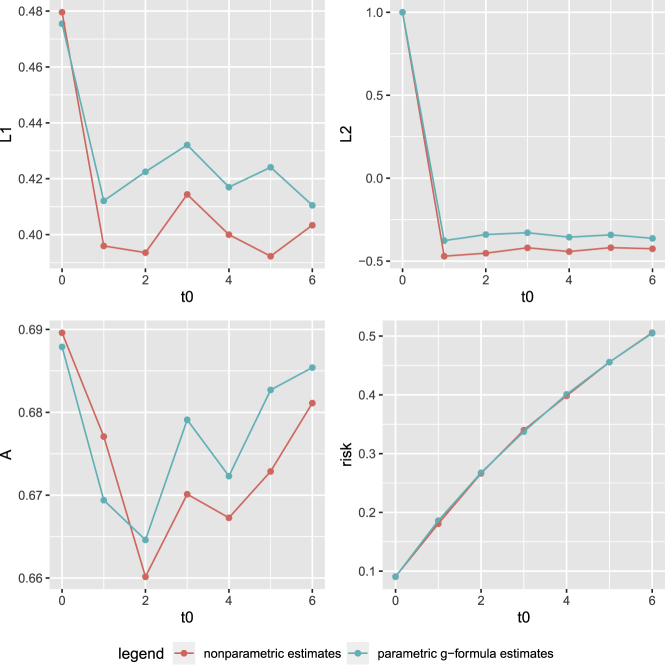
Comparison of the Nonparametric and Parametric g-formula Estimates of the Event Risk by Each Follow-Up Time, and Covariate Means, under the Natural Course

In this example, the plot(gform_basic) command additionally produces by default the plots shown in Figure 1. These plots compare the nonparametric and parametric g-formula estimates of the event risk by each follow-up time, and covariate means, under the natural course.

#### Example 2: Estimating the Effect of Threshold Interventions on the Mean of a Binary End of Follow-up Outcome

The example dataset binary_eofdata consists of 17,500 observations on 2,500 individuals with a maximum of 7 follow-up times. The outcome of interest corresponds to the indicator of an event only in the last interval K+1. The variables in the dataset are:

time: The time index

id_num: A unique identifier for each individual

cov1: A time-varying covariate; binary

cov2: A time-varying covariate; continuous

cov3: A baseline covariate; continuous

treat: The treatment variable; binary

outcome: The outcome of interest; continuous

In Box 5, we illustrate use of the function gformula to estimate the probability of experiencing the outcome in interval K+1=7 under a time-varying threshold intervention that maintains the treatment in all intervals at or above 1, as well as under “never treat” and the natural course. We also illustrate the construction of 95% confidence intervals using, for simplicity, 20 bootstrap samples, as well as parallelization of the bootstrapping and estimation procedures across the total number of available CPU cores minus 1 (see Appendix C.4 for details concerning bootstrapping and parallelization). When increasing the number of bootstrap samples to 500 in this example, the program took 32 min to run on a MacBook Air with i5 (1.6 GHz) and 8 GB of RAM when using R version 3.6.2.

Box 5 > outcome_type <- 'binary_eof'

> id <- 'id_num'

> time_name <- 'time'

> covnames <- c('cov1', 'cov2', 'treat')

> outcome_name <- 'outcome'

> histories <- c(lagged, cumavg)

> histvars <- list(c('treat', 'cov1', 'cov2'), c('cov1', 'cov2'))

> covtypes <- c('binary', 'zero-inflated normal', 'normal')

> covparams <- list(covmodels = c(cov1 ∼ lag1_treat + lag1_cov1 + lag1_cov2 +

+  cov3 + time,

+  cov2 ∼ lag1_treat + cov1 + lag1_cov1 +

+  lag1_cov2 + cov3 + time,

+  treat ∼ lag1_treat + cumavg_cov1 +

+  cumavg_cov2 + cov3 + time))

> ymodel <- outcome ∼ treat + cov1 + cov2 + lag1_cov1 + lag1_cov2 + cov3

> intvars <- list('treat', 'treat')

> interventions <- list(list(c(static, rep(0, 7))),

+  list(c(threshold, 1, Inf)))

> int_descript <- c('Never treat', 'Threshold - lower bound 1')

> nsimul <- 10000

> ncores <- parallel::detectCores() – 1

> gform_bin_eof <- gformula(obs_data = binary_eofdata,

+  outcome_type = outcome_type, id = id,

+  time_name = time_name, covnames = covnames,

+  outcome_name = outcome_name, covtypes = covtypes,

+  covparams = covparams, ymodel = ymodel,

+  intvars = intvars, interventions = interventions,

+  int_descript = int_descript, histories = histories,

+  histvars = histvars, basecovs = c("cov3"),

+  seed = 1234, parallel = TRUE, nsamples = 20,

+  nsimul = nsimul, ncores = ncores)

> gform_bin_eof

PREDICTED RISK UNDER MULTIPLE INTERVENTIONS

Intervention  Description

0  Natural course

1  Never treat

2  Threshold - lower bound 1

Sample size = 2500, Monte Carlo sample size = 10000

Number of bootstrap samples = 20

Reference intervention = natural course (0)

k Interv. NP mean g-form mean Mean SE Mean lower 95% CI Mean upper 95% CI

6  0  0.0988  0.09864823  0.005971232  0.08966186  0.1088847

6  1  NA  0.09285333  0.009144334  0.08112626  0.1095961

6  2  NA  0.08771817  0.015160129  0.06744307  0.1186075

Mean ratio  MR SE  MR lower 95% CI  MR upper 95% CI  Mean difference

1.0000000  0.00000000  1.0000000  1.000000  0.000000000

0.9412569  0.08428133  0.7867005  1.093676  −0.005794899

0.8892016  0.15300028  0.6345815  1.186092  −0.010930062

MD SE  MD lower 95% CI  MD upper 95% CI

0.000000000  0.00000000  0.000000000

0.008528051  −0.02271965  0.009143285

0.015406542  −0.03892206  0.018154694

The output reports parametric g-formula estimates of the mean outcome (g-form mean) under “never treat” (0.093), the threshold intervention (0.088), and the natural course (0.099). It also reports nonparametric estimates of the outcome mean (NP mean) under the natural course (0.099), along with 95% confidence intervals for these means and mean differences and ratios comparing each intervention with the natural course.

#### Example 3: Estimating the Effect of Treatment Strategies on the Mean of a Continuous End of Follow-Up Outcome

The example dataset continuous_eofdata again consists of 7,500 observations on 2,500 individuals with a maximum of 7 follow-up times, where the outcome corresponds to a characteristic only in the last interval (e.g., systolic blood pressure in interval 7). The variables in the dataset are:

t0: The time index

id: A unique identifier for each individual

L1: A time-varying covariate; categorical

L2: A time-varying covariate; continuous

L3: A baseline covariate; continuous

A: The treatment variable; binary

Y: The outcome of interest; continuous

In Box 6, we illustrate how to estimate the mean outcome at K+1=7 under “never treat” versus “always treat.” We also illustrate in this example how to include a restricted cubic spline function of a variable in a model statement using the rcspline.eval function from the **Hmisc** package.

Box 6 > library("Hmisc")

> outcome_type <- 'continuous_eof'

> id <- 'id'

> time_name <- 't0'

> covnames <- c('L1', 'L2', 'A')

> outcome_name <- 'Y'

> covtypes <- c('categorical', 'normal', 'binary')

> histories <- c(lagged)

> histvars <- list(c('A', 'L1', 'L2'))

> covparams <- list(covmodels = c(L1 ∼ lag1_A + lag1_L1 + L3 + t0 +

+  rcspline.eval(lag1_L2, knots = c(-1, 0, 1)),

+  L2 ∼ lag1_A + L1 + lag1_L1 + lag1_L2 + L3 + t0,

+  A ∼ lag1_A + L1 + L2 + lag1_L1 + lag1_L2 + L3 + t0))

> ymodel <- Y ∼ A + L1 + L2 + lag1_A + lag1_L1 + lag1_L2 + L3

> intvars <- list('A', 'A')

> interventions <- list(list(c(static, rep(0, 7))),

+  list(c(static, rep(1, 7))))

> int_descript <- c('Never treat', 'Always treat')

> nsimul <- 10000

> gform_cont_eof <- gformula(obs_data = continuous_eofdata,

+  outcome_type = outcome_type, id = id,

+  time_name = time_name, covnames = covnames,

+  outcome_name = outcome_name, covtypes = covtypes,

+  covparams = covparams, ymodel = ymodel,

+  intvars = intvars, interventions = interventions,

+  int_descript = int_descript, histories = histories,

+  histvars = histvars, basecovs = c("L3"),

+  nsimul = nsimul, seed = 1234)

> gform_cont_eof

PREDICTED RISK UNDER MULTIPLE INTERVENTIONS

Intervention  Description

0  Natural course

1  Never treat

2  Always treat

Sample size = 2500, Monte Carlo sample size = 10000

Number of bootstrap samples = 0

Reference intervention = natural course (0)

k  Interv.  NP mean  g-form mean  Mean ratio  Mean difference

6  0  −4.414543  −4.348234  1.000000  0.0000000

6  1  NA  −3.107835  0.714735  1.2403990

6  2  NA  −4.603006  1.058592  −0.2547717

## Discussion

The **gfoRmula** package provides an implementation of the parametric g-formula in R for estimating the effects of sustained treatment strategies using longitudinal data with time-varying treatment and confounders. The package handles survival outcomes with or without competing events as well as end of follow-up outcomes. It provides flexible options for estimating the required conditional covariate distributions and outcome means in realistic settings with many follow-up times and high-dimensional confounders. It allows for joint interventions on multiple time-varying treatments and flexible intervention rules. It also has the ability to incorporate *a priori* deterministic knowledge of covariate distributions when available (Appendix C.5).

The parametric g-formula based on joint density modeling (as described here) is one of several estimators of the g-formula functions. Other approaches considered in Appendix A.3 are iterative expectation estimators,^24^ inverse probability weighted estimators,^25^, ^26^, ^27^ and doubly robust approaches.^24^^,^^28^ All these approaches are nonparametrically equivalent, but they may give different estimates in realistic settings with some degree of model misspecification.

These different methods have different advantages and disadvantages in practice. The parametric g-formula may more naturally accommodate complex treatment strategies but the validity of its estimates relies on the correct specification of a larger number of models. On the other hand, the parametric g-formula based on joint density modeling, unlike the other estimators, has the ability to incorporate knowledge of confounder distributions when available.

However, the relative performance of the parametric g-formula based on joint density modeling compared with other estimators has not been thoroughly studied. In addition to providing a flexible tool for estimating effects of sustained treatment strategies from observational data, the **gfoRmula** package can facilitate simulation studies in R of the relative performance of this method compared with other estimators under different data-generating mechanisms.

## Experimental Procedures

Full experimental procedures are provided in Supplemental Information.

## Data and Code Availability

The **gfoRmula** package is freely available online. The released version of the package is available on the Comprehensive R Archive Network (CRAN) at https://CRAN.R-project.org/package=gfoRmula, and the development version of the package is available on GitHub at https://github.com/CausalInference/gfoRmula. The package is under the GNU General Public License version 3.

The dataset and code used in the benchmark presented in Appendix D are available at https://github.com/CausalInference/gfoRmula-benchmark and are under the MIT Licence.

## Supplemental Information

Document S1. Supplemental Experimental Procedures

Document S2. Article plus Supplemental Information
