# Supplementary material for: gfoRmula: An R Package for Estimating the Effects of Sustained Treatment Strategies via the Parametric g-formula
Source: Patterns (N Y). 2020 May 18;1(3):100008. doi: 10.1016/j.patter.2020.100008 (PMC7351102; doi:10.1016/j.patter.2020.100008)
Supplement: Document S2. Article plus Supplemental Information [file mmc2.pdf]

Patterns

Descriptor

gfoRmula: An R Package for Estimating the Effects of Sustained Treatment Strategies via the Parametric g-formula

Highlights

- gfoRmula supports survival outcomes and binary/continuous end of follow-up outcomes
- gfoRmula has several options for handling competing events
- Users can specify custom covariate models, history functions, and interventions
- Users can incorporate deterministic knowledge and visit processes

Authors

Sean McGrath, Victoria Lin, Zilu Zhang, Lucia C. Petito, Roger W. Logan, Miguel A. Hernán, Jessica G. Young

Correspondence

sean_mcgrath@g.harvard.edu

In Brief

McGrath et al. present the statistical software package, gfoRmula. This package implements the parametric g-formula, a statistical method to estimate the causal effects of sustained treatment strategies from observational data with time-varying treatments, confounders, and outcomes.

McGrath et al., 2020, Patterns *1*, 100008
June 12, 2020 © 2020 The Author(s).
https://doi.org/10.1016/j.patter.2020.100008

CellPress

Patterns

CellPress
OPEN ACCESS

Descriptor

gfoRmula: An R Package for Estimating the Effects of Sustained Treatment Strategies via the Parametric g-formula

Sean McGrath,[1,8,9,*] Victoria Lin,[2,8] Zilu Zhang,[3,4] Lucia C. Petito,[5] Roger W. Logan,[6] Miguel A. Hernán,[1,6,7] and Jessica G. Young[3]
[1]Department of Biostatistics, Harvard T.H. Chan School of Public Health, Boston, MA 02115, USA
[2]School of Computer Science, Carnegie Mellon University, Pittsburgh, PA 15213, USA
[3]Department of Population Medicine, Harvard Medical School and Harvard Pilgrim Health Care Institute, Boston, MA 02215, USA
[4]Department of Medical Oncology, Dana Farber Cancer Institute, Boston, MA 02215, USA
[5]Feinberg School of Medicine, Northwestern University, Chicago, IL 60611, USA
[6]Department of Epidemiology, Harvard T.H. Chan School of Public Health, Boston, MA 02115, USA
[7]Harvard-MIT Division of Health Sciences and Technology, Cambridge, MA 02139, USA
[8]These authors contributed equally
[9]Lead Contact
*Correspondence: sean_mcgrath@g.harvard.edu
https://doi.org/10.1016/j.patter.2020.100008

THE BIGGER PICTURE Causal inference is a core task of data science. When data from randomized experiments are not available, data analysts often rely on nonexperimental (observational) data to estimate causal effects. The parametric g-formula is a statistical method to estimate the causal effects of sustained treatment strategies from observational data with time-varying treatments, confounders, and outcomes. Although this methodology was introduced in the 1980s, it has not been widely used due to the lack of open-source software. This article presents the gfoRmula package, an implementation of the parametric g-formula in R. The aim of this software is to facilitate the application of the parametric g-formula to complex, observational data to answer causal questions. Furthermore, this package helps provide a way to compare the performance of the parametric g-formula to other methods in the causal inference literature.

1 2 3 4 5 **Proof-of-Concept:** Data science output has been formulated, implemented, and tested for one domain/problem

SUMMARY

Researchers are often interested in estimating the causal effects of sustained treatment strategies, i.e., of (hypothetical) interventions involving time-varying treatments. When using observational data, estimating those effects requires adjustment for confounding. However, conventional regression methods cannot appropriately adjust for confounding in the presence of treatment-confounder feedback. In contrast, estimators derived from Robins's g-formula may correctly adjust for confounding even if treatment-confounder feedback exists. The package gfoRmula implements in R one such estimator: the parametric g-formula. This estimator can be used to estimate the effects of binary or continuous time-varying treatments as well as contrasts defined by static or dynamic, deterministic, or random interventions, as well as interventions that depend on the natural value of treatment. The package accommodates survival outcomes as well as binary or continuous outcomes measured at the end of follow-up. This paper describes the gfoRmula package, along with motivating background, features, and examples.

INTRODUCTION

Researchers are often interested in using longitudinal data to estimate the causal effects of hypothetical interventions that are sustained over time (equivalently, "treatment strategies," "rules," and "policies") on a population outcome mean. Standard regression methods for confounding control generally fail to recover such causal effects, which involve time-varying treatments, when time-varying confounders are themselves affected by past treatment.[1] For example, in studies of the effect of time-varying

This is an open access article under the CC BY license (http://creativecommons.org/licenses/by/4.0/).

antiretroviral treatment strategies on long-term mortality risk in HIV-positive individuals, CD4 cell count is a time-varying confounder, i.e., at follow-up time k it is a risk factor for both future treatment and mortality. It is also, itself, affected by past treatment.

In such settings, alternative estimators derived from Robins's "g-formula" may recover effects of sustained treatment interventions. The g-formula characterized by a user-specified treatment strategy is a function of the observed longitudinal data. Under untestable assumptions, including that sufficient covariates are measured to adjust for confounding,[2] this data function equals the outcome mean/risk had that intervention been implemented in all individuals in the study population. The package **gfoRmula** (current version 0.3.1) implements in `R` one estimator of the g-formula: "the parametric g-formula,"[3,4] available at https://CRAN.R-project.org/package=gfoRmula. In general, the g-formula is a high-dimensional sum or integral over a function of (1) the observed outcome mean (or, in survival settings, time-varying hazards) conditional on each observed treatment and confounder history, (2) the observed joint distribution of the confounders at each time k conditional on each observed treatment and confounder history, and (3) an intervention density defining the time-varying user-specified treatment strategy. The parametric g-formula estimates (1) and (2) via parametric models and approximates the sum via a Monte Carlo simulation.

The **gfoRmula** package accommodates general interventions or treatment strategies, which may be "static" or "dynamic"[4–10] and, further, "deterministic" or "random."[11–15] It also may be used to estimate effects of treatment strategies that depend on the "natural value of treatment" at k; the value of treatment that would have been observed at k were the intervention discontinued directly before k.[16] The package also allows: (1) binary or continuous/multilevel time-varying treatments; (2) different types of outcomes (survival or continuous/binary end of follow-up); (3) data with competing events (survival outcomes) and loss to follow-up; (4) joint interventions on multiple treatments and other options.

The **gfoRmula** R package has many of the capabilities of the **GFORMULA** SAS macro,[17] as well some overlapping capabilities described for the **gformula** command in Stata,[18] to implement the parametric g-formula. However, the **gfoRmula** `R` package will facilitate use of this approach for many researchers who may only be able (or prefer) to conduct data analysis in `R` given its free and open-source (non-proprietary) platform. The **gfoRmula** `R` package also more easily allows users to incorporate their own helper functions in different steps of the algorithm.

In this article, we describe input dataset requirements and core features of the package along with various code examples. Theoretical background including formal definition of the observed data structure, causal estimands, and identifying assumptions, along with details of the estimation algorithm and some advanced package features, are provided in appendices.

RESULTS

In this section, we describe how to use the **gfoRmula** package and provide complete coding examples.

Using the gfoRmula Package

Specifying the Outcome Type

The main function in the **gfoRmula** package is the `gformula` function. The argument `outcome_type` specifies the type of the outcome of interest; `'survival'` should be used for survival outcomes (e.g., an indicator of all-cause mortality by an end of follow-up time $K + 1$); `'continuous_eof'` should be used for fixed continuous end of follow-up outcomes (e.g., blood pressure at time $K + 1$); and `'binary_eof'` should be used for fixed binary end of follow-up outcomes (e.g., indicator of high cognitive score at $K + 1$). For survival outcomes, the package outputs estimates of contrasts in failure risks by a specified follow-up time $k \leq K+1$ (i.e., the probability of failing by that time) under different user-specified treatment strategies. For end of follow-up outcomes, it outputs estimates of contrasts in the outcome mean at $K + 1$.

Required Structure of the Input Dataset

The input dataset to the `gformula` function must be an `R data.table`. For all outcome types, the input dataset should contain one record for each follow-up time k for each subject present at baseline (specified by the parameter `id`). The time index k must start at 0 (indexing baseline) and increase in increments of 1. Each column of the dataset will index one of p time-varying covariates $Z_{j,k}$, $j = 1, \ldots, p$. Additional columns will contain values of time-fixed baseline confounders (e.g., race, sex) with values repeated on each line k for that subject in the dataset.

In some cases, additional records (rows) may be specified containing pre-baseline information. If no records before baseline are needed to control for confounding, the parameter `time_name` is an index that specifies the follow-up time k, i.e., the value of `time_name` for each subject starts at 0 and increases in increments of 1 until, at most, K. If records before baseline are needed, `time_name` can specify the index of the pre-baseline time and the follow-up time k. Letting i denote the maximum number of pre-baseline times contained in the data, the value of `time_name` for each subject starts at $-i$ and increases in increments of 1 until, at most, K. Each subject can then have a maximum of $K+1+i$ records with $K + 1$, the end of follow-up of interest and $i=0$ when no additional pre-baseline records are included. See Appendix C.1 for additional coding specifications in the case of $i \neq 0$. In the remainder of the text, we presume $i=0$ unless otherwise specified.

A subject who is censored in interval $k+1$ $(C_k = 0, C_k + 1 = 1)$ will have $k+1$ records with time index k on the last line. Other requirements are dependent on the outcome type, which we describe below.

Survival Outcomes. In this case, the dataset should additionally contain a column Y_{k+1} indicating on line k whether the event of interest occurred by the next interval. A subject who first has the event of interest in interval $k+1$ will have only $k+1$ follow-up records with time index k on the last line. If $C_{k+1} = 1$ then the user has the choice to code Y_{k+1} as either `NA` or 0. The user does not need to include the column with a time-varying censoring indicator in the dataset. For additional notes on construction of the input dataset when there is censoring, including the choice of how to code Y_{k+1} and why a censoring indicator column is not needed, see the end of Appendix B.1.

If the event of interest is subject to a competing event for some subjects and some k and the user chooses to treat competing events as censoring events, competing events should be treated

CellPress
OPEN ACCESS

like any component of C_{k+1} and coding requirements above must be applied. In turn, in this case, the input dataset does not require a variable with competing event status. If, alternatively, competing events are not treated as censoring events, the user must include in the input dataset a column with a time-varying indicator of the competing event D_{k+1}. If $D_{k+1} = 1$ on a line k for a given subject, that subject will only have $k+1$ lines in the follow-up data with follow-up time k on the last line and, on that line, Y_{k+1} must be coded `NA`. See the datasets `basicdata_nocomp` and `basicdata` included with the package for examples of input datasets for survival outcomes without and with competing events, respectively.

When competing events are treated as censoring events, the algorithm targets a particular direct effect, i.e., a contrast of failure risks under different treatment strategies and an additional intervention that (somehow) eliminates competing events. Otherwise, the algorithm targets a particular total effect, i.e., this contrast but without elimination of competing events. The total effect is precisely a contrast of different (counterfactual) cause-specific cumulative incidences as defined in the classical survival analysis literature.[19,20] For details see Young et al.[21] and Appendix A.

The user can specify the parameter `time_points` to be a follow-up interval $k<K+1$ (with $K+1$ the maximum number of records for an individual in the dataset) in order to compute the risk estimates through $k<K+1$. The default value of `time_points` is $K+1$.

End of Follow-Up Outcomes. In this case, the dataset should additionally contain a column Y with the value of the outcome in interval $K+1$. Only the value of this variable on line $k=K$ is used in the algorithm. Death, if present, must be treated as a censoring event. For subjects who are first censored in interval $K+1$, these subjects will have $K+1$ follow-up records in the data, and Y on line $k=K$ must be coded as `NA`. For subjects censored prior to interval $K+1$, the value of Y will be ignored in the estimation algorithm. See the dataset `continuous_eofdata` included with the package for an example of an input dataset for an end of follow-up outcome.

Specifying the Input Dataset, Individual Identifier, Time Index, Outcome, and Competing Events

The input dataset is specified by the argument `obs_data`. The column of this dataset containing the individual identifier is specified by the argument `id`, the time index by the argument `time_name`, the outcome by the argument `outcome_name`, and, if present and not defined as a censoring event, competing event status by the argument `compevent_name`. Sample syntax for a survival outcome is given by:

```
gformula(..., outcome_type = 'survival',
         obs_data = obs_data,
         id = 'ID',
         time_name = 't0',
         outcome_name = 'Y',
         compevent_name = 'D')
```

Specifying Time-Varying and Baseline Covariates

The time-varying covariates Z_k are defined by the vector argument `covnames`. Time-fixed baseline confounders are defined by the vector argument `basecovs`. Sample syntax is given by:

```
gformula(..., covnames = c('Z1', 'Z2', 'Z3'),
         basecovs = c('race','sex'))
```

Generating Covariate Histories

As detailed in Appendix B, the parametric g-formula estimates the g-formula indexed by a user-specified treatment strategy in two steps. In step 1, (i) the observed outcome mean (or, in survival settings, time-varying hazards) conditional on each observed treatment and confounder history and (ii) the observed joint distribution of the confounders at each follow-up time k conditional on each observed treatment and confounder history are estimated. The joint distribution is estimated as a product of conditional distributions based on an arbitrarily ordered factorization of the p components of Z_k (see Appendix B for additional details and example below). In step 2, a Monte Carlo simulation is conducted in which the covariates are iteratively simulated at each follow-up time k using these estimates, where the simulated value of treatment is replaced with the value consistent with the user-specified treatment strategy.

The arguments `histvars` and `histories` must be used to specify any desired functions of history that will be used for estimation in step 1 (and then, in turn, in the simulation in step 2) of the estimation algorithm that cannot be defined within a model statement in `R`. For example, consider a time-varying covariate $Z_{4,k}$ named `Z4` in the input dataset and suppose the user assumes that the distributions of "future" covariates ordered under this arbitrary factorization of the vector Z_k, $Z_{j,k}$, $j>4$, depend on the history of $Z_{4,k}$ through the cumulative average of this variable through each k; i.e., $\frac{1}{k}\sum_{t=0}^{k} Z_{4,t}$ for $k>0$ and $Z_{4,0}$ otherwise. The user must then use `histvars` and `histories` to create in the dataset `obs_data` this additional time-varying covariate containing the cumulative average of $Z_{4,k}$ at each k.

Pre-coded functions of history for a covariate named `Zj` in the input dataset include:

- `lagged`: this adds a variable to the specified input dataset named `lagi_Zj`, containing the ith lag of `Zj` relative to the current follow-up time k (i.e., its value at $k-i$ for $k \geq i$) for all $i = 1, \ldots, r$ with r the desired number of lags. `lagi_Zj` is set to 0 on lines with $k<i$.
- `cumavg`: this adds a variable to the specified input dataset named `cumavg_Zj`, which contains the cumulative average of `Zj` up until the current follow-up time $k>0$. It is set to `Zj` at $k=0$.
- `lagavg`: this adds a variable to the specified input dataset named `lag_cumavgi_Zj` which contains the ith lag of the cumulative average of `Zj` relative to the current follow-up time k, $i = 1, \ldots, r$. `lag_cumavgi_Zj` is set to 0 on lines with $k<i$.

Note the desired number of lags r is specified using the argument `covmodels` (see the next section, Specifying Covariate Distributions).

The package will apply the function of history listed in the qth element of the vector argument `histories` to all variables listed in the qth element of the list argument `histvars`. Therefore, the length of `histvars` and `histories` need to match. Sample syntax that would add lagged, cumulative average, and lagged cumulative average functions of the covariates `Z1`, `Z2`, and `Z3` as new variables to the input dataset `obs_data` for use in estimation of conditional distributions/hazards/means (see the

Box 1.

```
gformula(..., covnames = c('Z1', 'Z2', 'Z3'),
         histories = c(lagged, cumavg, lagavg),
         histvars = list(c('Z1', 'Z2', 'Z3'), c('Z1', 'Z2', 'Z3'),
                         c('Z1', 'Z2', 'Z3')))
```

sections Specifying Covariate Distributions and Specifying Outcome and Competing Event Models) is shown in Box 1.

Throughout this subsection, it was assumed that data on time-varying covariates at pre-baseline are either not available or not necessary to adjust for confounding. See Appendix C.1 for how these history functions are applied when additional records containing pre-baseline values of time-varying covariates are included in the input dataset.

Specifying Covariate Distributions

The vector argument `covtypes` is used to specify the distributions of each time-varying covariate $Z_{j,k}$ conditional on a function of history $(Z_{j-1,k},\ldots,Z_{1,k},\overline{Z}_{k-1})$, $j = 1,\ldots,p$, and $k = 0,\ldots,K$, how parameters of those distributions will be estimated, and, in turn, how covariate values in step 2 of the algorithm will be simulated. The **gfoRmula** package supplies a number of pre-programmed options for input to the argument `covtypes`, which are described in detail in Appendix C.2: `'binary'`, `'normal'`, `'categorical'`, `'bounded normal'`, `'zero-inflated normal'`, `'truncated normal'`, `'absorbing'`, and `'categorical time'`. Each of the elements of `covtypes` requires its own set of specific sub-parameters, which are contained within the list argument `covparams`. Each sub-parameter vector within this list must be the same length as `covnames` and `covtypes`. Several examples are also given later in the Examples section.

Specifying Outcome and Competing Event Models

The argument `ymodel` takes as input an `R` model statement. For survival outcomes, this model statement is passed to `glm` in `R` with `binomial` family and `logit` link to estimate the hazard at each follow-up time k conditional on history (i.e., $p_k(\bar{l}_k,\bar{a}_k)$ from step 1.b of the algorithm in Appendix B.1). Therefore, this model should generally depend on a function of k.

For continuous end of follow-up outcomes, this model statement is passed to `glm` with `gaussian` family and `identity` link to estimate the mean of the outcome at end of follow-up conditional on time-varying and baseline covariate history (i.e., $\mu(\bar{l}_K,\bar{a}_K)$ from modified step 2.c of the algorithm in Appendix B.2). Therefore, this model is not dependent on follow-up time k. Similarly, for binary end of follow-up outcomes, this model statement is passed to `glm` with `binomial` family and `logit` link to estimate the mean of the outcome at end of follow-up conditional on history (which is not dependent on k).

The argument `compevent_model` takes as input an `R` model statement passed to `glm` in `R` with `binomial` family and `logit` link to estimate the (cause-specific) hazard of the competing event at each follow-up time k conditional on history (i.e., $q_k(\bar{l}_k,\bar{a}_k)$ from step 1.c of the algorithm in Appendix B.1). The model is generally dependent on k. This argument should only be specified for survival outcomes and when the user chooses not to define the competing event as a censoring event. Several syntax examples are given in Examples.

Specifying the Interventions

We define an "intervention rule" as a strategy involving an intervention on one or more treatments. However, we will use the terms "intervention," "intervention rule," and "strategy" interchangeably with "joint interventions" referring to strategies with interventions on more than one treatment.

The arguments `intvars`, `interventions`, and `int_times` are jointly used to define the user-specified treatment interventions to be compared. See Appendix A.2 for a review of classification of interventions (static versus dynamic, deterministic versus random).

`intvars` is a list of vectors. The number of vector elements of this list should be the number of user-specified interventions of interest. Each vector element specifies the time-varying covariates to be intervened upon under that intervention (i.e., the treatments/exposures). This vector will have a single element if the intervention involves intervening on only a single time-varying covariate (we will call this a "single intervention") and will have multiple elements if the intervention involves intervening on multiple time-varying covariates (a "joint intervention").

`interventions` is a list whose elements are lists of vectors. We refer to the list specified for `interventions` as the "outer list" and refer to the elements of this outer list as the "inner lists". The length of the outer list should be the number of user-specified interventions of interest. Each inner list specifies one or more treatment strategies, each one defined by a vector of arguments. The inner list will include only a single vector for an element corresponding to a single treatment strategy and will include multiple vectors for an element corresponding to a joint intervention (each vector element specifying the intervention for a given covariate under that intervention). The first element of a vector specifying an intervention for a given covariate must be the function that defines the intervention, and the following elements specify the input parameter(s) of the intervention function (if applicable). Box 2 includes a list of pre-coded interventions or treatment strategies available with the package with examples (see Examples for additional examples).

To specify the intervention used as the "reference," i.e., the denominator in the risk/mean ratio/difference calculations, set `ref_int` equal to the index of interventions in which the desired reference intervention is specified. By default, `ref_int = 0`, or the natural course.

Optionally, users can specify the time points in which interventions are applied via the `int_times` argument. As with the `intvars` argument, `int_times` is a list whose elements are lists of vectors. Each vector specifies the time points at which the relevant intervention is applied on the corresponding variable

CellPress
OPEN ACCESS

Box 2.

- `static`: the function `static` specifies a deterministic static intervention. It requires specification of a vector of length `time_points` with the *k*th element the value of treatment to be assigned under that intervention at follow-up time *k*. The following is sample syntax to compare two static interventions on a binary time-varying covariate `A` that sets this variable to 0 at all follow-up times versus 1 at all follow-up times:

```
gformula(..., time_points = 10,
         intvars = list(c('A'), c('A')),
         interventions = list(list(c(static, rep(0, 10))),
                              list(c(static, rep(1, 10)))))
```

The following extends this example to compare joint static interventions that sets two time-varying covariates `A1` and `A2` to 0 at all follow-up times versus 1 at all follow-up times:

```
gformula(..., time_points = 10,
         intvars = list(c('A1', 'A2'), c('A1', 'A2')),
         interventions = list(list(c(static, rep(0, 10)),
                                   c(static, rep(0, 10))),
                              list(c(static, rep(1, 10)),
                                   c(static, rep(1, 10)))))
```

- `threshold`: the function `threshold` specifies a threshold intervention, an example of an intervention at *k* that depends on the natural value of treatment *k* (see Appendix A.2). Under a threshold intervention, at each follow-up time *k*, the treatment value is set to the simulated (natural) value of treatment at *k* if it falls within a user-specified range and, otherwise, the treatment value under intervention is set to the maximum of that range (if the simulated value is above) or the minimum of that range (if the simulated value is below).[13,22,23] This treatment strategy requires specification of the minimum and maximum of the desired range. If no maximum is desired this should be set to `Inf` and if no minimum is desired this should be set to `-Inf`. The following is sample syntax to compare two threshold interventions maintaining a time-varying covariate `Z2` at all follow-up times at $\geq$2 versus $\geq$3:

```
gformula(..., intvars = list(c('Z2'), c('Z2')),
         interventions = list(list(c(threshold, 2, Inf)),
                              list(c(threshold, 3, Inf))))
```

- `natural`: the function `natural` specifies the natural course intervention under which the simulated value is assigned at each follow-up time *k*. Risks/means under the natural course are always computed by default; thus, this intervention does not need to be specified.

in `intvars`. When an intervention is not applied at a follow-up time *k* (not included in `int_times`), the simulated (natural) value is used. By default, all interventions are applied at all follow-up time points. In Box 3, the extension of the above example compares two interventions on the time-varying covariate `Z2` under which the natural value of treatment is assigned at $k = 0, 1$ and, for $k \geq 2$, a threshold intervention is applied that ensures the treatment is assigned values $\geq$2 versus $\geq$3.

Output

The `gformula` function returns an S3 class object (of class `gformula`), which is a list that contains the following main elements: (1) a `data.table` containing the nonparametric estimates of the natural course risk/mean outcome (see Appendix B) and the parametric g-formula estimates of the risk/mean outcome under each specified intervention at each follow-up time point; (2) the coefficients, standard errors, variance-covariance matrices of the parameters of the fitted models, and RMSE values of the models fit in step 1 of the algorithm; and optionally, (3) the fitted models (e.g., `glm` objects) in step 1 of the algorithm and (4) the simulated histories from step 2 of the algorithm under each specified intervention. Objects returned by the `gformula` function also have class `gformula_survival` (for survival outcomes), `gformula_continuous_eof` (for continuous end of follow-up outcomes), or `gformula_binary_eof` (for binary end of follow-up outcomes).

`S3` `print`, `summary`, `plot`, `coef`, and `vcov` methods are available for objects returned by the `gformula` function. The `plot` method plots parametric g-formula versus nonparametric estimates of the risks by each follow-up interval under the natural course intervention for `gformula_survival`-type objects, along with parametric versus nonparametric estimates of covariate means under the natural course for `gformula_survival`,

Box 3.

```
gformula(..., time_points=10,
         intvars = list(c('Z2'), c('Z2')),
         interventions = list(list(c(threshold, 2, Inf)),
                              list(c(threshold, 3, Inf))),
         int_times = list(list(2:9), list(2:9)))
```

Box 4.

```
> outcome_type <- 'survival'
> id <- 'id'
> time_points <- 7
> time_name <- 't0'
> covnames <- c('L1', 'L2', 'A')
> outcome_name <- 'Y'
> covtypes <- c('binary', 'bounded normal', 'binary')
> histories <- c(lagged, lagavg)
> histvars <- list(c('A', 'L1', 'L2'), c('L1', 'L2'))
> covparams <- list(covmodels = c(L1 ~ lag1_A + lag_cumavg1_L1 + lag_cumavg1_L2 +
+                                   L3 + t0,
+                                 L2 ~ lag1_A + L1 + lag_cumavg1_L1 +
+                                   lag_cumavg1_L2 + L3 + t0,
+                                 A ~ lag1_A + L1 + L2 + lag_cumavg1_L1 +
+                                   lag_cumavg1_L2 + L3 + t0))
> ymodel <- Y ~ A + L1 + L2 + L3 + lag1_A + lag1_L1 + lag1_L2 + t0
> intvars <- list('A', 'A')
> interventions <- list(list(c(static, rep(0, time_points))),
+                       list(c(static, rep(1, time_points))))
> int_descript <- c('Never treat', 'Always treat')
> nsimul <- 10000
> gform_basic <- gformula(obs_data = basicdata_nocomp,
+                         outcome_type = outcome_type, id = id,
+                         time_points = time_points, time_name = time_name,
+                         covnames = covnames, outcome_name = outcome_name,
+                         covtypes = covtypes, covparams = covparams,
+                         ymodel = ymodel, intvars = intvars,
+                         interventions = interventions,
+                         int_descript = int_descript, histories = histories,
+                         histvars = histvars, basecovs = c('L3'),
+                         nsimul = nsimul, seed = 1234)
> gform_basic
PREDICTED RISK UNDER MULTIPLE INTERVENTIONS
Intervention    Description
0               Natural course
1               Never treat
2               Always treat
Sample size = 2500, Monte Carlo sample size = 10000
Number of bootstrap samples = 0
Reference intervention = natural course (0)
k    Interv.    NP risk    g-form risk    Risk ratio    Risk difference
6    0          0.5056     0.5048280      1.0000000     0.0000000
6    1          NA         0.7314631      1.4489355     0.2266352
6    2          NA         0.2339747      0.4634741     -0.2708533
```

gformula_continuous_eof, and gformula_binary_eof-type objects. See also Young et al.[10]

Examples

In this subsection, we provide complete examples along with a description of output.

Example 1: Estimating the Effect of Static Treatment Strategies on Risk of a Failure Event

The example dataset basicdata_nocomp consists of 13,170 observations on 2,500 individuals with a maximum of 7 follow-up times. No individuals are censored in this dataset. The variables in the dataset are:

t0: The time index
id: A unique identifier for each individual
L1: A time-varying covariate; binary
L2: A time-varying covariate; continuous
L3: A baseline covariate; continuous
A: The treatment variable; binary
Y: The outcome/failure event of interest; time-varying indicator of failure

Data for the first subject, who survives the whole follow-up, is presented below:

	id	t0	L1	L2	L3	A	Y
1:	1	0	0	1.1470920	5	1	0
2:	1	1	0	−0.9254032	5	1	0
3:	1	2	0	−0.9899824	5	0	0
4:	1	3	1	1.0057421	5	1	0
5:	1	4	1	−1.1956468	5	1	0
6:	1	5	0	−0.9697723	5	1	0
7:	1	6	1	−1.0887002	5	1	0

The subject identifier `id` does not have to be a number as long as it is unique for each individual.

The syntax in Box 4 can be used to estimate, using this dataset, the risk of failure from the event of interest by $K+1=7$ under a strategy that sets treatment to 0 at all follow-up times for all individuals ("never treat") and a strategy that sets it to 1 at all times ("always treat"). Note that we must use the `nsimul` parameter to set the number of simulated histories (s, Appendix B) because in this example the baseline sample size is $n<10,000$.

In this example, the parametric g-formula estimates of the risk (`g-form risk`) by end of follow-up under "never treat," "always treat," and the natural course is 0.731, 0.234, and 0.505, respectively. The risk ratio by $K+1$ comparing "never treat" with the natural course is 1.45. The nonparametric estimate (`NP risk`) of the natural course risk is 0.506 and close to the parametric g-formula estimate of this risk. This supports (but does not guarantee) the absence of gross model misspecification.[10]

In this example, the `plot(gform_basic)` command additionally produces by default the plots shown in Figure 1. These plots compare the nonparametric and parametric g-formula estimates of the event risk by each follow-up time, and covariate means, under the natural course.

Example 2: Estimating the Effect of Threshold Interventions on the Mean of a Binary End of Follow-up Outcome

The example dataset `binary_eofdata` consists of 17,500 observations on 2,500 individuals with a maximum of 7 follow-up times. The outcome of interest corresponds to the indicator of an event only in the last interval $K+1$. The variables in the dataset are:

`time`: The time index
`id_num`: A unique identifier for each individual
`cov1`: A time-varying covariate; binary
`cov2`: A time-varying covariate; continuous
`cov3`: A baseline covariate; continuous
`treat`: The treatment variable; binary
`outcome`: The outcome of interest; continuous

In Box 5, we illustrate use of the function `gformula` to estimate the probability of experiencing the outcome in interval $K+1=7$ under a time-varying threshold intervention

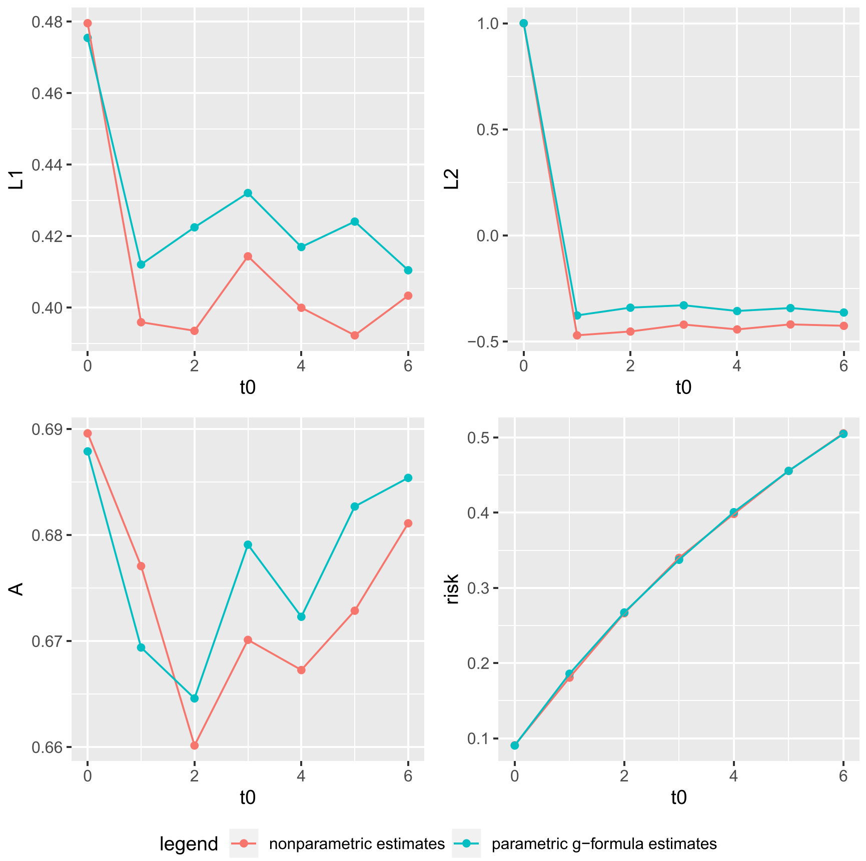

Figure 1. Comparison of the Nonparametric and Parametric g-formula Estimates of the Event Risk by Each Follow-Up Time, and Covariate Means, under the Natural Course

Box 5.

```
> outcome_type <- 'binary_eof'
> id <- 'id_num'
> time_name <- 'time'
> covnames <- c('cov1', 'cov2', 'treat')
> outcome_name <- 'outcome'
> histories <- c(lagged, cumavg)
> histvars <- list(c('treat', 'cov1', 'cov2'), c('cov1', 'cov2'))
> covtypes <- c('binary', 'zero-inflated normal', 'normal')
> covparams <- list(covmodels = c(cov1 ~ lag1_treat + lag1_cov1 + lag1_cov2 +
+                                   cov3 + time,
+                                 cov2 ~ lag1_treat + cov1 + lag1_cov1 +
+                                   lag1_cov2 + cov3 + time,
+                                 treat ~ lag1_treat + cumavg_cov1 +
+                                   cumavg_cov2 + cov3 + time))
> ymodel <- outcome ~ treat + cov1 + cov2 + lag1_cov1 + lag1_cov2 + cov3
> intvars <- list('treat', 'treat')
> interventions <- list(list(c(static, rep(0, 7))),
+                       list(c(threshold, 1, Inf)))
> int_descript <- c('Never treat', 'Threshold - lower bound 1')
> nsimul <- 10000
> ncores <- parallel::detectCores() - 1
> gform_bin_eof <- gformula(obs_data = binary_eofdata,
+                           outcome_type = outcome_type, id = id,
+                           time_name = time_name, covnames = covnames,
+                           outcome_name = outcome_name, covtypes = covtypes,
+                           covparams = covparams, ymodel = ymodel,
+                           intvars = intvars, interventions = interventions,
+                           int_descript = int_descript, histories = histories,
+                           histvars = histvars, basecovs = c("cov3"),
+                           seed = 1234, parallel = TRUE, nsamples = 20,
+                           nsimul = nsimul, ncores = ncores)
> gform_bin_eof
PREDICTED RISK UNDER MULTIPLE INTERVENTIONS
Intervention      Description
0                 Natural course
1                 Never treat
2                 Threshold - lower bound 1
Sample size = 2500, Monte Carlo sample size = 10000
Number of bootstrap samples = 20
Reference intervention = natural course (0)
k    Interv.    NP mean    g-form mean    Mean SE        Mean lower 95% CI    Mean upper 95% CI
6    0          0.0988     0.09864823     0.005971232    0.08966186           0.1088847
6    1          NA         0.09285333     0.009144334    0.08112626           0.1095961
6    2          NA         0.08771817     0.015160129    0.06744307           0.1186075
Mean ratio     MR SE         MR lower 95% CI    MR upper 95% CI    Mean difference
1.0000000      0.00000000    1.0000000          1.000000           0.000000000
0.9412569      0.08428133    0.7867005          1.093676           −0.005794899
0.8892016      0.15300028    0.6345815          1.186092           −0.010930062
MD SE          MD lower 95% CI    MD upper 95% CI
0.000000000    0.00000000         0.000000000
0.008528051    −0.02271965        0.009143285
0.015406542    −0.03892206        0.018154694
```

that maintains the treatment in all intervals at or above 1, as well as under "never treat" and the natural course. We also illustrate the construction of 95% confidence intervals using, for simplicity, 20 bootstrap samples, as well as parallelization of the bootstrapping and estimation procedures across the total number of available CPU cores minus 1 (see Appendix C.4

CellPress
OPEN ACCESS

Box 6.

```
> library("Hmisc")
> outcome_type <- 'continuous_eof'
> id <- 'id'
> time_name <- 't0'
> covnames <- c('L1', 'L2', 'A')
> outcome_name <- 'Y'
> covtypes <- c('categorical', 'normal', 'binary')
> histories <- c(lagged)
> histvars <- list(c('A', 'L1', 'L2'))
> covparams <- list(covmodels = c(L1 ~ lag1_A + lag1_L1 + L3 + t0 +
+                                  rcspline.eval(lag1_L2, knots = c(-1, 0, 1)),
+                                L2 ~ lag1_A + L1 + lag1_L1 + lag1_L2 + L3 + t0,
+                                A ~ lag1_A + L1 + L2 + lag1_L1 + lag1_L2 + L3 + t0))
> ymodel <- Y ~ A + L1 + L2 + lag1_A + lag1_L1 + lag1_L2 + L3
> intvars <- list('A', 'A')
> interventions <- list(list(c(static, rep(0, 7))),
+                       list(c(static, rep(1, 7))))
> int_descript <- c('Never treat', 'Always treat')
> nsimul <- 10000
> gform_cont_eof <- gformula(obs_data = continuous_eofdata,
+                            outcome_type = outcome_type, id = id,
+                            time_name = time_name, covnames = covnames,
+                            outcome_name = outcome_name, covtypes = covtypes,
+                            covparams = covparams, ymodel = ymodel,
+                            intvars = intvars, interventions = interventions,
+                            int_descript = int_descript, histories = histories,
+                            histvars = histvars, basecovs = c("L3"),
+                            nsimul = nsimul, seed = 1234)
> gform_cont_eof
PREDICTED RISK UNDER MULTIPLE INTERVENTIONS
Intervention     Description
0                Natural course
1                Never treat
2                Always treat
Sample size = 2500, Monte Carlo sample size = 10000
Number of bootstrap samples = 0
Reference intervention = natural course (0)
k    Interv.    NP mean      g-form mean    Mean ratio    Mean difference
6    0          −4.414543    −4.348234      1.000000      0.0000000
6    1          NA           −3.107835      0.714735      1.2403990
6    2          NA           −4.603006      1.058592      −0.2547717
```

for details concerning bootstrapping and parallelization). When increasing the number of bootstrap samples to 500 in this example, the program took 32 min to run on a MacBook Air with i5 (1.6 GHz) and 8 GB of RAM when using `R` version 3.6.2.

The output reports parametric g-formula estimates of the mean outcome (`g-form mean`) under "never treat" (0.093), the threshold intervention (0.088), and the natural course (0.099). It also reports nonparametric estimates of the outcome mean (`NP mean`) under the natural course (0.099), along with 95% confidence intervals for these means and mean differences and ratios comparing each intervention with the natural course.

Example 3: Estimating the Effect of Treatment Strategies on the Mean of a Continuous End of Follow-Up Outcome

The example dataset `continuous_eofdata` again consists of 7,500 observations on 2,500 individuals with a maximum of 7 follow-up times, where the outcome corresponds to a characteristic only in the last interval (e.g., systolic blood pressure in interval 7). The variables in the dataset are:

`t0`: The time index
`id`: A unique identifier for each individual
`L1`: A time-varying covariate; categorical
`L2`: A time-varying covariate; continuous
`L3`: A baseline covariate; continuous

A: The treatment variable; binary

Y: The outcome of interest; continuous

In Box 6, we illustrate how to estimate the mean outcome at $K+1=7$ under ‘‘never treat’’ versus ‘‘always treat.’’ We also illustrate in this example how to include a restricted cubic spline function of a variable in a model statement using the `rcspline.eval` function from the **Hmisc** package.

DISCUSSION

The **gfoRmula** package provides an implementation of the parametric g-formula in R for estimating the effects of sustained treatment strategies using longitudinal data with time-varying treatment and confounders. The package handles survival outcomes with or without competing events as well as end of follow-up outcomes. It provides flexible options for estimating the required conditional covariate distributions and outcome means in realistic settings with many follow-up times and high-dimensional confounders. It allows for joint interventions on multiple time-varying treatments and flexible intervention rules. It also has the ability to incorporate *a priori* deterministic knowledge of covariate distributions when available (Appendix C.5).

The parametric g-formula based on joint density modeling (as described here) is one of several estimators of the g-formula functions. Other approaches considered in Appendix A.3 are iterative expectation estimators,[24] inverse probability weighted estimators,[25–27] and doubly robust approaches.[24,28] All these approaches are nonparametrically equivalent, but they may give different estimates in realistic settings with some degree of model misspecification.

These different methods have different advantages and disadvantages in practice. The parametric g-formula may more naturally accommodate complex treatment strategies but the validity of its estimates relies on the correct specification of a larger number of models. On the other hand, the parametric g-formula based on joint density modeling, unlike the other estimators, has the ability to incorporate knowledge of confounder distributions when available.

However, the relative performance of the parametric g-formula based on joint density modeling compared with other estimators has not been thoroughly studied. In addition to providing a flexible tool for estimating effects of sustained treatment strategies from observational data, the **gfoRmula** package can facilitate simulation studies in R of the relative performance of this method compared with other estimators under different data-generating mechanisms.

EXPERIMENTAL PROCEDURES

Full experimental procedures are provided in Supplemental Information.

DATA AND CODE AVAILABILITY

The **gfoRmula** package is freely available online. The released version of the package is available on the Comprehensive R Archive Network (CRAN) at https://CRAN.R-project.org/package=gfoRmula, and the development version of the package is available on GitHub at https://github.com/CausalInference/gfoRmula. The package is under the GNU General Public License version 3.

The dataset and code used in the benchmark presented in Appendix D are available at https://github.com/CausalInference/gfoRmula-benchmark and are under the MIT Licence.

SUPPLEMENTAL INFORMATION

Supplemental Information can be found online at https://doi.org/10.1016/j.patter.2020.100008.

ACKNOWLEDGMENTS

The authors thank L. Paloma Rojas Saunero, Eleanor Murray, Sara Lodi, and Louisa Smith for testing of the package. This work was funded by National Institutes of Health grant R37 AI102634. S.M. acknowledges support from the National Science Foundation Graduate Research Fellowship Program under grant DGE1745303, National Library of Medicine of the National Institutes of Health under award number T32LM012411, and Fonds de recherche du Québec - Nature et technologies B1X research scholarship. Any opinions, findings, and conclusions or recommendations expressed in this material are those of the author(s) and do not necessarily reflect the views of the National Science Foundation, National Institutes of Health, or Fonds de recherche du Québec.

AUTHOR CONTRIBUTIONS

M.A.H. and J.G.Y. conceived the project. S.M., V.L., Z.Z., L.C.P., R.W.L., M.A.H., and J.G.Y. developed the presented software. S.M., V.L., and J.G.Y. drafted the manuscript. All authors contributed to and approved the final version of the manuscript.

DECLARATION OF INTERESTS

The authors declare no competing interests.

Received: December 18, 2019
Revised: January 30, 2020
Accepted: February 24, 2020
Published: May 18, 2020

REFERENCES

1. Robins, J.M., and Hernán, M.A. (2009). Estimation of the causal effects of time-varying exposures. In Advances in Longitudinal Data Analysis, G. Fitzmaurice, M. Davidian, G. Verbeke, and G. Molenberghs, eds. (Chapman; Hall/CRC Press), pp. 553–600.
2. Robins, J.M. (1986). A new approach to causal inference in mortality studies with a sustained exposure period: application to the healthy worker survivor effect, [Errata (1987) in Computers and Mathematics with Applications 14, 917-921. Addendum (1987) in Computers and Mathematics with Applications 14, 923-945. Errata (1987) to addendum in Computers and Mathematics with Applications 18, 477.]. Math. Model. 7, 1393–1512.
3. Lin, V., McGrath, S., Zhang, Z., Logan, R.W., Petito, L.C., Young, J.G., and Hernán, M.A. (2019). gfoRmula: parametric g-formula. R package version 0.3.0. https://CRAN.Rproject.org/package=gfoRmula.
4. Hernán, M.A., and Robins, J. (2020). Causal Inference: What If (Chapman & Hall/CRC).
5. Orellana, L., Rotnitzky, A., and Robins, J.M. (2010a). Dynamic regime marginal structural mean models for estimation of optimal dynamic treatment regimes, part I: main content. Int. J. Biostat. *6*, https://doi.org/10.2202/1557-4679.1200.
6. Orellana, L., Rotnitzky, A., and Robins, J.M. (2010b). Dynamic regime marginal structural mean models for estimation of optimal dynamic treatment regimes, part II: proofs and additional results. Int. J. Biostat. *6*, https://doi.org/10.2202/1557-4679.1242.

CellPress
OPEN ACCESS

7. van der Laan, M.J., Petersen, M.L., and Joffe, M.M. (2005). History-adjusted marginal structural models and statically-optimal dynamic treatment regimens. Int. J. Biostat. *1*, https://doi.org/10.2202/1557-4679.1003.
8. Murphy, S.A., van der Laan, M.J., and Robins, J.M. (2001). Marginal mean models for dynamic regimes. J. Am. Stat. Assoc. *96*, 1410–1423.
9. Cain, L.E., Robins, J.M., Lanoy, E., Logan, R., Costagliola, D., and Hernán, M.A. (2010). When to start treatment? a systematic approach to the comparison of dynamic regimes using observational data. Int. J. Biostat. *6*, https://doi.org/10.2202/1557-4679.1212.
10. Young, J.G., Cain, L.E., Robins, J.M., O'Reilly, E.J., and Hernán, M.A. (2011). Comparative effectiveness of dynamic treatment regimes: an application of the parametric g-formula. Stat. Biosci. *3*, 119–143.
11. Díaz Muñoz, I., and van der Laan, M.J. (2012). Population intervention causal effects based on stochastic interventions. Biometrics *68*, 541–549.
12. Haneuse, S., and Rotnitzky, A. (2013). Estimation of the effect of interventions that modify the received treatment. Stat. Med. *32*, 5260–5277.
13. Young, J.G., Hernán, M.A., and Robins, J.M. (2014). Identification, estimation and approximation of risk under interventions that depend on the natural value of treatment using observational data. Epidemiol. Meth. *3*, 1–19.
14. Young, J.G., Logan, R.W., Robins, J.M., and Hernán, M.A. (2019). Inverse probability weighted estimation of risk under representative interventions in observational studies. J. Am. Stat. Assoc. *114*, 938–947.
15. Kennedy, E. (2019). Nonparametric causal effects based on incremental propensity score interventions. J. Am. Stat. Assoc. *114*, 645–656.
16. Richardson, T.S., and Robins, J.M. (2013). Single World Intervention Graphs (SWIGs): A Unification of the Counterfactual and Graphical Approaches to Causality (Center for Statistics and the Social Sciences, University of Washington Series).
17. Logan, R., Young, J., Taubman, S., Lodi, S., Picciotto, S., Danaei, G., and Hernán, M.A. (2016). GFORMULA SAS MACRO. https://www.hsph.harvard.edu/causal/software.
18. Daniel, R., De Stavola, B., and Cousens, S. (2011). gformula: estimating causal effects in the presence of time-varying confounding or mediation using the g-computation formula. Stata J. *11*, 479–517.
19. Fine, J., and Gray, R. (1999). A proportional hazards model for the subdistribution of a competing risk. J. Am. Stat. Assoc. *94*, 496–509.
20. Geskus, R.B. (2016). Data Analysis with Competing Risks and Intermediate States (Chapman; Hall/CRC).
21. Young, J., Stensrud, M., Tchetgen Tchetgen, E., and Hernán, M. (2020). A causal framework for classical statistical estimands in failure time settings with competing events. Stat. Med. https://doi.org/10.1002/sim.8471.
22. Robins, J.M., Hernán, M.A., and Siebert, U. (2004). Effects of multiple interventions. In Comparative Quantification of Health Risks: Global and Regional Burden of Disease Attributable to Selected Major Risk Factors, M. Ezzati, A.D. Lopez, A. Rodgers, and C.J.L. Murray, eds. (World Health Organization), pp. 2191–2231.
23. Taubman, S.L., Robins, J.M., Mittleman, M.A., and Hernán, M.A. (2009). Intervening on risk factors for coronary heart disease: an application of the parametric g-formula. Int. J. Epidemiol. *38*, 1599–1611.
24. Bang, H., and Robins, J.M. (2005). Doubly robust estimation in missing data and causal inference models. Biometrics *61*, 692–972.
25. Hernán, M.A., Brumback, B., and Robins, J.M. (2000). Marginal structural models to estimate the causal effect of zidovudine on the survival of HIV-positive men. Epidemiology *11*, 561–570.
26. Robins, J.M., Hernán, M.A., and Brumback, B. (2000). Marginal structural models and causal inference in epidemiology. Epidemiology *11*, 550–560.
27. Robins, J.M. (1998). Marginal structural models. In 1997 Proceedings of the American Statistical Association, Section on Bayesian Statistical Science (American Statistical Association), pp. 1–10.
28. Lendle, S.D., Schwab, J., Petersen, M.L., and van der Laan, M.J. (2017). ltmle: an R package implementing targeted minimum loss-based estimation for longitudinal data. J. Stat. Softw. *81*, 1–21.

PATTER, Volume *1*

Supplemental Information

gfoRmula: An R Package for Estimating the Effects of Sustained Treatment Strategies via the Parametric g-formula

Sean McGrath, Victoria Lin, Zilu Zhang, Lucia C. Petito, Roger W. Logan, Miguel A. Hernán, and Jessica G. Young

A Background

A.1 Notation and observed data structure

We consider a longitudinal study where measurements of treatment(s), and covariates are regularly updated on each of $i = 1, \ldots, n$ subjects over a specified follow-up period. Let $k = 0, \ldots, K+1$ denote fixed follow-up intervals (e.g. months) with baseline measurements taken in interval $k = 0$ and interval $K+1$ corresponding to the specified end of follow-up (e.g. 60 months).

In each interval k, assume the following are measured: Let A_k be a treatment variable (or vector of treatment variables) measured in interval k (e.g. an indicator of antiretroviral treatment initiation by interval k; minutes of physical activity in interval k) and L_k a vector of time-varying covariates (e.g., CD4 cell count, BMI) assumed to precede A_k with L_0 possibly including time-fixed baseline covariates (e.g., race, baseline age). In clinical cohorts, including studies based on electronic medical records, L_k may be defined in terms of last measured values of a covariate relative to interval k. For example, in a clinical cohort of HIV-infected patients, CD4 cell count is not measured every month. It is only measured in a month when the patient comes for a visit. In this case L_k may be defined in terms of the last measured value of CD4 cell count relative to month k. Further, L_k may contain an indicator of whether that last measured value is current or not (i.e. if a visit at k has occurred).[1]

Let $Y \equiv Y_{K+1}$ denote the outcome of interest. Y may correspond to either a fixed (continuous or binary) end of follow-up outcome *in* interval $K+1$ (e.g. blood pressure *in* interval $K+1$; an indicator of obesity *in* interval $K+1$) or a survival outcome; i.e., an indicator of failure from an event of interest *by* $K+1$ (e.g. an indicator of stroke *by* $K+1$). For survival outcomes, we implicitly include in L_k an indicator of failure from the event of interest by earlier $k < K+1$ (Y_k). Past values of outcomes at the end of follow-up may also be components of L_k (e.g. when the outcome is obesity status at $K+1$, obesity at any $k < K+1$ may be a component of L_k). Note that "end of follow-up" may correspond to any user-specified follow-up of interest for which data is available and need not correspond to the administrative end of the study (e.g. the user may specify $K+1$ as 10 month follow-up even if the administrative end of the study was 60 months).

Let C_k denote an indicator of censoring and D_k an indicator of a competing event (for a survival outcome) or truncation by death (for an end of follow-up outcome) by interval k, respectively. When a survival outcome is not subject to competing events (e.g. all-cause mortality), we define $D_k \equiv 0$ for all k. For end of follow-up outcomes, the user must define D_k as an implicit component of C_k (i.e. as a censoring event). For survival outcomes, the user may choose to define D_k as an implicit component of C_k or L_k. Under the latter choice, competing events are not treated as censoring events. See Young et al.[2] as well as below regarding how this choice impacts the interpretation of the analysis and estimation algorithms.

We denote the history of a random variable using overbars; for example, $\overline{A}_k = (A_0, \ldots, A_k)$ is the observed treatment history through interval k. By notational convention, we set $\overline{L}_{-1}$ and $\overline{A}_{-1}$ to be identically 0.

A.2 Causal effect definitions

In each interval k, we generally define an intervention on A_k that may, at most, depend on past measured variables as a random draw from an intervention density $f^{int}(a_k|\bar{l}_k, \overline{a}_{k-1}, \overline{C}_k = 0)$ with $(\overline{a}_k, \bar{l}_k)$ a possible realization of $(\overline{A}_k, \overline{L}_k)$.

We classify an intervention $f^{int}(a_k|\bar{l}_k, \overline{a}_{k-1}, \overline{C}_k = 0)$ on A_k as *deterministic* if $f^{int}(a_k|\bar{l}_k, \overline{a}_{k-1}, \overline{Y}_k = 0)$ may only equal zero or one for all k and $(\overline{a}_k, \bar{l}_k)$; otherwise we classify it as *random*. We may further classify the intervention as *static* if $f^{int}(a_k|\bar{l}_k, \overline{a}_{k-1}, \overline{C}_k = 0)$ does not depend on $\bar{l}_k$ for any k; otherwise we classify it as *dynamic*. See Young et al.[3,4], as well as Section 2.1.8 and Appendix C.9, for examples.

Beyond interventions of the form $f^{int}(a_k|\bar{l}_k, \overline{a}_{k-1}, \overline{C}_k = 0)$, we will also define an intervention on A_k that, in addition to past measured variables, depends on the natural value of treatment at k. We define such an intervention as a random draw from an alternative intervention density $f^{d}(a_k|a_k^*, \bar{l}_k, \overline{a}_{k-1}, \overline{C}_k = 0)$ where a_k^* is any value in the support of A_k which, in the observational study, coincides with the natural value of treatment at k.[3,5]

Our goal is to estimate the causal effect on the mean of Y (which corresponds to the risk of the event of interest by $K+1$ in the case of survival outcomes) had, contrary to fact we implemented two different hypothetical interventions on A_k at all $k=0,\dots,K$ in the study population where these interventions may be any user-specified choices of either $f^{int}(a_k|\bar{l}_k,\overline{a}_{k-1},\overline{C}_k=0)$ or $f^d(a_k|a_k^*,\bar{l}_k,\overline{a}_{k-1},\overline{C}_k=0)$. Implicit in the definition of all interventions is "abolish censoring throughout follow-up" or "Set $C_k=0$ at all k". By this, how the user chooses to define C_k will impact the interpretation of the estimand.[6] For survival outcomes, when D_k is defined as an implicit component of C_k, this effect is a special case of a direct effect that does not capture any treatment effect on the competing event; otherwise it is a special case of a total effect that may capture these effects.[2]

A.3 Identifying assumptions and the g-formula

Let $g=(g_0,...g_K)$ be a *deterministic* regime, strategy, or intervention (static or dynamic) on A_k that depends, at most, on the measured covariate and treatment history, characterized by the intervention density $f^{int}(a_k|\bar{l}_k,\overline{a}^g_{k-1},\overline{C}_k=0)=I(a_k=a^g_k)$ that also eliminates censoring, where $a^g_s=g_s\left(\bar{\ell}_s,\overline{a}^g_{s-1}\right)$ is any component of $\overline{a}^g_k=(a^g_0,\dots,a^g_k)$ and $\overline{a}^g_s$ is recursively defined by the function g_s of $(\bar{l}_s,\overline{a}^g_{s-1})$, $s=0,\dots,k$.

Define Y^g and $\overline{L}^g_K$ as the outcome and covariate histories, respectively, for an individual in the study population had, possibly contrary to fact, his/her treatment been assigned according to a deterministic regime g. Further, let $\mathcal{G}$ be the *set of all deterministic interventions* g on A_k (both static and dynamic). Following Robins[7], the mean of Y had all subjects been assigned treatment according to $f^{int}(a_k|\bar{l}_k,\overline{a}_{k-1},\overline{C}_k=0)$ and had censoring been eliminated is equivalent to $\sum_{g\in\mathcal{G}}w(g)\text{E}[Y^g]$ where $w(g)$ is a $g-$specific weight defined in terms of the choice of $f^{int}(a_k|\bar{l}_k,\overline{a}_{k-1},\overline{Y}_k=0)$. See also the appendix of Young et al.[3]

We now define three g-specific identifying conditions:

1. Exchangeability: For all $k=0,\dots,K$

$$Y^g\coprod(A_k,C_{k+1})|\overline{L}_k=\bar{l}_k,\overline{A}_{k-1}=\overline{a}^g_{k-1},C_k=0 \tag{1}$$

   The exchangeability condition (1) is expected to hold in an experiment where the treatment A_k and censoring C_{k+1} are physically randomized at each k possibly depending on treatment and covariate history $(\overline{L}_k,\overline{A}_{k-1})$. This condition is not, however, guaranteed to hold in an observational study, or in a randomized trial with censoring, and cannot be empirically examined. Exchangeability is sometimes referred to as the assumption of "no unmeasured confounding" and $\overline{L}_k$ the "measured confounder history through k".

2. Positivity:

$$\begin{aligned}&f_{\overline{A}_{k-1},\overline{L}_k,C_k}(\overline{a}^g_{k-1},\bar{l}_k,0)\neq 0 \implies\\&\Pr[C_{k+1}=0|\overline{L}_k=\bar{l}_k,\overline{A}_k=\overline{a}^g_k,C_k=0)\times\\&f^{obs}(a^g_k|\overline{a}^g_{k-1},\bar{l}_k,\overline{C}_k=0)>0\ w.p.1.\end{aligned} \tag{2}$$

   where $f^{obs}(a^g_k|\overline{a}^g_{k-1},\bar{l}_k,\overline{C}_k=0)\equiv f_{A_k|\overline{A}_{k-1},\overline{L}_k,\overline{C}_k}(a^g_k|\overline{a}^g_{k-1},\bar{l}_k,\bar{0})$

3. Consistency: If $\overline{A}_K=\overline{a}^g_K$ and $\overline{C}_{K+1}=0$ then $Y=Y^g$ and $\overline{L}_K=\overline{L}^g_K$.

Given the three conditions above hold for all $g\in\mathcal{G}$ such that positivity holds when we replace the observed treatment density $f^{obs}(a^g_k|\overline{a}^g_{k-1},\bar{l}_k,\overline{C}_k=0)$ with the intervention density $f^{int}(a^g_k|\overline{a}^g_{k-1},\bar{l}_k,\overline{C}_k=0)$, then $\sum_{g\in\mathcal{G}}w(g)\text{E}[Y^g]$ is equivalent to the g-formula characterized by $f^{int}(a_k|\bar{l}_k,\overline{a}_{k-1},\overline{C}_k=0)$ (see

Robins[7]):

$$\sum_{\overline{a}_K}\sum_{\overline{l}_K}\mathsf{E}[Y|\overline{L}_K=\overline{l}_K,\overline{A}_K=\overline{a}_K,\overline{C}_K=0]\times$$
$$\prod_{j=0}^{K}\{f(l_j|\overline{l}_{j-1},\overline{a}_{j-1},\overline{C}_j=0)f^{int}(a_j|\overline{l}_j,\overline{a}_{j-1},\overline{C}_j=0)\} \quad (3)$$

where $f(l_k|\overline{l}_{k-1},\overline{a}_{k-1},\overline{C}_k=0)$ is the observed joint density of the confounders at k conditional on treatment and covariate history, and remaining uncensored, through k. We use a summation symbol in (3) and elsewhere for notational simplicity. However, in general, when $(\overline{A}_k,\overline{L}_k)$ contains any continuous components, sums would be replaced with integrals.

When interest is in a survival outcome, expression (3) is the g-formula for risk of the event of interest by $K+1$ under intervention $f^{int}(a_k|\overline{l}_k,\overline{a}_{k-1},\overline{C}_k=0)$ which, pulling the implicit prior survival indicator Y_k out of L_k, can be more explicitly written as:

$$\sum_{\overline{a}_K}\sum_{\overline{l}_K}\sum_{k=0}^{K}\Pr[Y_{k+1}=1|\overline{L}_k=\overline{l}_k,\overline{A}_k=\overline{a}_k,\overline{C}_{k+1}=\overline{Y}_k=0]\times$$
$$\prod_{j=0}^{k}\{f(l_j|\overline{l}_{j-1},\overline{a}_{j-1},\overline{C}_j=0)f^{int}(a_j|\overline{l}_j,\overline{a}_{j-1},\overline{C}_j=0)$$
$$\Pr[Y_j=0|\overline{L}_{j-1}=\overline{l}_{j-1},\overline{A}_{j-1}=\overline{a}_{j-1},\overline{C}_j=\overline{Y}_{j-1}=0]\} \quad (4)$$

As above, when the survival outcome is subject to competing events, we may choose to treat competing events (D_k) as an implicit component of C_k or L_k, $k=0,\ldots,K+1$. Under the former, expression (4) will not depend on the distribution of competing events. Under the latter, it will depend on this distribution; in this case, we can more explicitly write expression (4) as

$$\sum_{\overline{a}_K}\sum_{\overline{l}_K}\sum_{k=0}^{K}\Pr[Y_{k+1}=1|\overline{L}_k=\overline{l}_k,\overline{A}_k=\overline{a}_k,\overline{C}_{k+1}=\overline{D}_{k+1}=\overline{Y}_k=0]\times$$
$$\prod_{j=0}^{k}\{f(l_j|\overline{l}_{j-1},\overline{a}_{j-1},\overline{C}_j=\overline{D}_j=\overline{Y}_j=0)f^{int}(a_j|\overline{l}_j,\overline{a}_{j-1},\overline{C}_j=\overline{Y}_j=0)$$
$$\Pr[Y_j=0|\overline{L}_{j-1}=\overline{l}_{j-1},\overline{A}_{j-1}=\overline{a}_{j-1},\overline{C}_j=\overline{D}_j=\overline{Y}_{j-1}=0]$$
$$\Pr[D_{j+1}=0|\overline{L}_j=\overline{l}_j,\overline{A}_j=\overline{a}_j,\overline{C}_{j+1}=\overline{D}_j=\overline{Y}_j=0]\} \quad (5)$$

See Young et al.[2] for details.

Conditions required for identification based on only observed variables of the mean/risk of Y under an alternative intervention characterized by $f^d(a_k|\overline{a}^*_k,\overline{l}_k,\overline{a}_{k-1},\overline{C}_k=0)$, which additionally depends on the history of the natural value of treatment, are generally stronger than those defined above. We refer the reader elsewhere for details of these conditions.[3,5] Provided these stronger conditions hold, the mean of Y under an intervention characterized by $f^d(a_k|\overline{a}^*_k,\overline{l}_k,\overline{a}_{k-1},\overline{C}_k=0)$ is equivalent to the extended g-formula of Robins et al.[8],

$$\sum_{\overline{a}^*_K}\sum_{\overline{a}_K}\sum_{\overline{l}_K}\mathsf{E}[Y|\overline{L}_K=\overline{l}_K,\overline{A}_K=\overline{a}_K,\overline{C}_K=0]\times$$
$$\prod_{j=0}^{K}\{f(l_j|\overline{l}_{j-1},\overline{a}_{j-1},\overline{C}_j=0)f^d(a_j|a^*_j,\overline{l}_j,\overline{a}_{j-1},\overline{C}_j=0)$$
$$f(a^*_j|\overline{l}_j,\overline{a}_{j-1},\overline{C}_j=0)\} \quad (6)$$

where $f(a_j^*|\bar{l}_j, \bar{a}_{j-1}, \overline{C}_j = 0)$ is the observed treatment density conditional on the measured past evaluated at some possibly realized values $(\overline{A}_j, \overline{L}_j) = (a_j^*, \bar{a}_{j-1}, \bar{l}_j)$.

In motivating the general estimation algorithm described in the next section, it is useful to consider a generic version of the g-formula for the mean/risk of the outcome characterized by either an intervention density $f^d(a_k|\bar{a}_k^*, \bar{l}_k, \bar{a}_{k-1}, \overline{C}_k = 0)$ which depends on the natural value of treatment at k or $f^{int}(a_k|\bar{l}_k, \bar{a}_{k-1}, \overline{C}_k = 0)$ which depends, at most, on the measured past treatment and confounder history. For $Z_k = (L_k, A_k)$ and realization $z_k = (l_k, a_k^*)$, $k = 0, \ldots, K$ we have :

$$\sum_{\bar{a}_K} \sum_{\bar{z}_K} \mathsf{E}[Y|\overline{L}_K = \bar{l}_K, \overline{A}_K = \bar{a}_K, \overline{C}_K = 0] \times \prod_{j=0}^{K} \{f(z_j|\bar{l}_{j-1}, \bar{a}_{j-1}, \overline{C}_j = 0) h^{user}(\bar{a}_j, a_j^*, \bar{l}_j)\} \tag{7}$$

is algebraically equivalent to (6) when $h^{user}(\bar{a}_k, a_k^*, \bar{l}_k)$ is chosen as some $f^d(a_k|\bar{a}_k^*, \bar{l}_k, \bar{a}_{k-1}, \overline{C}_k = 0)$ and algebraically equivalent to (3) when $h^{user}(\bar{a}_k, a_k^*, \bar{l}_k)$ is alternatively chosen as some $f^{int}(a_k|\bar{l}_k, \bar{a}_{k-1}, \overline{C}_k = 0)$ as, in this latter case, the expression does not depend on the observed treatment density $f(a_k^*|\bar{l}_k, \bar{a}_{jk1}, \overline{C}_k = 0)$.[3]

Similarly, for survival outcomes, we can expressly write the g-formula for risk by $K+1$ under generic treatment interventions $h^{user}(\bar{a}_j, a_j^*, \bar{l}_j)$

$$\begin{aligned}\sum_{\bar{a}_K} \sum_{\bar{z}_K} \sum_{k=0}^{K} \Pr[&Y_{k+1} = 1|\overline{L}_k = \bar{l}_k, \overline{A}_k = \bar{a}_k, \overline{C}_{k+1} = \overline{D}_{k+1} = \overline{Y}_k = 0] \times \\ &\prod_{j=0}^{k} \{f(z_j|\bar{l}_{j-1}, \bar{a}_{j-1}, \overline{C}_j = \overline{D}_j = 0 = \overline{Y}_j = 0) h^{user}(\bar{a}_j, a_j^*, \bar{l}_j) \\ &\Pr[Y_j = 0|\overline{L}_{j-1} = \bar{l}_{j-1}, \overline{A}_{j-1} = \bar{a}_{j-1}, \overline{C}_j = \overline{D}_j = \overline{Y}_{j-1} = 0] \\ &\Pr[D_{j+1} = 0|\overline{L}_j = \bar{l}_j, \overline{A}_j = \bar{a}_j, \overline{C}_{j+1} = \overline{D}_j = \overline{Y}_j = 0]\}\end{aligned} \tag{8}$$

when the outcome is subject to competing events and competing events are not treated as censoring events. Following arguments above and in Young et al.[2], replacing $\Pr[D_{j+1} = 0|\overline{L}_j = \bar{l}_j, \overline{A}_j = \bar{a}_j, \overline{C}_{j+1} = \overline{D}_j = \overline{Y}_j = 0]$ with 1 for all j and $(\bar{a}_j, \bar{l}_j)$ would give the g-formula for risk by $K+1$ under an intervention $h^{user}(\bar{a}_j, a_j^*, \bar{l}_j)$ when competing events are treated as censoring events and, thus, under an intervention that eliminates competing events along with other possible forms of censoring.

B Estimation algorithm

B.1 Survival outcomes

The following describes the general computational algorithm for estimating the g-formula for risk of the outcome (the event of interest by $K+1$) under a user-specified intervention $h^{user}(\bar{a}_k, a_k^*, \bar{l}_k)$. This corresponds to (i) expression (8) when competing events are not treated as censoring events; (ii) a restricted version of (8) that replaces $\Pr[D_{j+1} = 0|\overline{L}_j = \bar{l}_j, \overline{A}_j = \bar{a}_j, \overline{C}_{j+1} = \overline{D}_j = \overline{Y}_j = 0]$ with 1 when competing events are treated as censoring events for all j (i.e. an implicit component of C_{j+1}) and $(\bar{a}_j, \bar{l}_j)$; and (iii) a restricted version of (8) with $D_k \equiv 0$ for all k when the outcome is not subject to competing events.

Let $Z_k = (L_k, A_k)$ such that for all $k = 0, \ldots, K$,

$$f(Z_k|\overline{L}_{k-1}, \overline{A}_{k-1}, \overline{C}_k = \overline{D}_k = \overline{Y}_k = 0) = \prod_{j=1}^{p} f(Z_{j,k}|Z_{j-1,k}, \ldots, Z_{1,k}, \overline{L}_{k-1}, \overline{A}_{k-1}, \overline{C}_k = \overline{D}_k = \overline{Y}_k = 0)$$

where $Z_{j,k}$ is the jth component of the vector Z_k, $j = 1, \ldots, p$ for a, generally, arbitrary permutation of these p components. Note that certain permutations will be preferred when *a priori* knowledge of the distributions of certain components of Z_k conditional on values of other components is known (see Appendix C.5).

For a user-chosen value of K, treatment rule $h^{user}(\overline{a}_k, a^*_k, \bar{l}_k)$ and permutation of Z_k, $k = 0, \ldots, K$, we apply the following algorithm to a subject-interval input data set constructed according to the instructions of Section 2.1.2:

1. Using all subject interval records:

   (a) If $k > 0$, estimate the conditional densities $f(Z_{j,k}|Z_{j-1,k}, \ldots, Z_{1,k}, \overline{L}_{k-1}, \overline{A}_{k-1}, \overline{C}_k = \overline{D}_k = \overline{Y}_k = 0)$ under a distributional assumption on $Z_{j,k}$ given "history" $(Z_{j-1,k}, \ldots, Z_{1,k}, \overline{L}_{k-1}, \overline{A}_{k-1})$, $j = 1, \ldots, p$. This distribution will be used to simulate a value of $Z_{j,k}$ in Step 2 at each $k > 0$. For example, dichotomous $Z_{j,k}$ will, by default, be generated from a Bernoulli distribution with mean $\mu(Z_{j-1,k}, \ldots, Z_{1,k}, \overline{L}_{k-1}, \overline{A}_{k-1}) = \Pr[Z_{j,k} = 1|Z_{j-1,k}, \ldots, Z_{1,k}, \overline{L}_{k-1}, \overline{A}_{k-1}, C_k = D_k = Y_k = 0]$. For $Z_{j,k}$ with many levels, the user can select among several pre-specified distributions (e.g. Normal) or can rely on a user-supplied distribution. The user has the option to estimate the conditional mean of $Z_{j,k}$ $\mu(Z_{j-1,k}, \ldots, Z_{1,k}, \overline{L}_{k-1}, \overline{A}_{k-1})$ for a specified function of the "history" $(Z_{j-1,k}, \ldots, Z_{1,k}, \overline{L}_{k-1}, \overline{A}_{k-1})$ using pre-specified fit options (e.g. generalized linear models with specified link function) or other user-supplied functions (e.g. generalized additive models, classification and regression trees) that produce a fitted object based on one line of data. Syntax for these specifications is described in Section 2.1.6 via specification of the parameters `covtypes` and `covparams` . These conditional mean models are pooled over time k and thus the user must specify the function of time k, if any, on which they will depend (see Appendices 2.1.6 and C.3). When the variance of $Z_{j,k}$ is not a function of the mean under the distributional assumption (e.g. when $Z_{j,k}$ is assumed Normal), the variance is automatically estimated by the model residual mean squared error.

   (b) Estimate the conditional probability (discrete hazard) of the event of interest at each time $k+1$ $k = 0, \ldots, K$ conditional on treatment and covariate history and surviving and remaining uncensored to the previous time, $p_k(\bar{l}_k, \overline{a}_k) \equiv \Pr[Y_{k+1} = 1|\overline{L}_k = \bar{l}_k, \overline{A}_k = \bar{a}_k, \overline{C}_{k+1} = \overline{D}_{k+1} = 0, \overline{Y}_k = 0]$. These estimates are automatically based on fitting a pooled over time logistic regression model with Y_{k+1} the dependent variable where the user specifies the function of "history" $(\overline{L}_k, \overline{A}_k)$ included as independent variables in the fit. See Section 2.1.7.

   (c) If the event of interest is subject to competing events and competing events are not defined as censoring events: Estimate the conditional probability (discrete hazard) of the competing event at each time k conditional on treatment and covariate history and surviving and remaining uncensored to the previous time $q_k(\bar{l}_k, \overline{a}_k) \equiv \Pr[D_{k+1} = 1|\overline{L}_k = \bar{l}_k, \overline{A}_k = \bar{a}_k, \overline{C}_{k+1} = \overline{D}_k = 0, \overline{Y}_k = 0]$. These estimates are automatically based on fitting a pooled over time logistic regression model with D_{k+1} the dependent variable where the user specifies the function of "history" $(\overline{L}_k, \overline{A}_k)$ and time included as independent variables in the fit. See Section 2.1.7.

2. Select a value $s \geq 10,000$, possibly such that $s = n$. If $s \neq n$ resample the original n ids s times with replacement and create a new data set where each possibly resampled record has a unique id $v = 1, \ldots, s$. Using the baseline covariate data from the original $v = 1, \ldots, n$ observations at baseline (if $s = n$) or the resampled observations (if $s \neq n$), for $k = 0, \ldots, K$ and $v = 1, \ldots, s$, do the following:

   (a) If $k = 0$, set $z_{0,v}$ to the observed values of Z_0 for id v. Otherwise, if $k > 0$, iteratively draw $z_{j,k,v}$ $j = 1, \ldots, p$ from estimates of the conditional densities $f(Z_{j,k}|Z_{j-1,k}, \ldots, Z_{1,k}, \overline{L}_{k-1}, \overline{A}_{k-1}, \overline{C}_k = \overline{D}_k = \overline{Y}_k = 0)$ (based on specifications from step 1.a) evaluated at $Z_{j,k} = z_{j,kv}$, $(Z_{1,k}, \ldots, Z_{j-1,k}, \overline{L}_{k-1}) = (z_{1,k,v}, \ldots, z_{j-1,k,v}, \bar{l}_{k-1,v})$, the previously drawn covariates and $\overline{A}_{k-1} = \overline{a}^{user}_{k-1,v}$, the previously assigned treatment through $k-1$ under the user-chosen strategy.

   (b) Denote the observed (at $k = 0$) or drawn (at $k > 0$) value(s) of treatment(s) in the vector $z_{k,v}$ as $a^*_{k,v}$. Assign $a^{user}_{k,v}$ according to the user specified rule $h^{user}(\overline{a}^{user}_{k,v}, a^*_k, \bar{l}_{k,v})$ which may or may

not depend on $a^*_{k,v}$. For example, suppose h^{user} is defined as the static deterministic strategy "always set treatment to the value 1." In this case, $a^{user}_{k,v}$ is always set to 1. Another example is "if the natural value of treatment at k is less than 30, set treatment to 30. Otherwise, do not intervene." In this case, the observed/drawn value of $a^*_{k,v}$ is examined. If $a^*_{k,v} < 30$ then $a^{user}_{k,v}$ is set to 30. Otherwise, $a^{user}_{k,v}$ is set to $a^*_{k,v}$. A third example is "no intervention on A_k" or the so-called *natural course* intervention. In this case, the intervention density h^{user} is defined as the observed treatment density and $a^{user}_{k,v}$ is always set to $a^*_{k,v}$. See Section 2.1.8 and Appendix C.9 for required syntax for specifying the treatment rules.

(c) Estimate $p_k(\bar{l}_{k,v}, \bar{a}^{user}_{k,v})$ based on specifications from step 1.b. Denote this estimate $\hat{p}(\bar{l}_{k,v}, \bar{a}^{user}_{k,v})$.

(d) If the event of interest is subject to competing events and competing events are not defined as censoring events: Estimate $q_k(\bar{l}_{k,v}, \bar{a}^{user}_{k,v})$ based on specifications from step 1.c. Denote this estimate $\hat{q}(\bar{l}_{k,v}, \bar{a}^{user}_{k,v})$.

3. Compute the intervention risk estimate as

$$\frac{1}{n}\sum_{v=1}^{n}\sum_{k=0}^{K}\hat{p}(\bar{a}^{user}_{k,v}, \bar{l}_{k,v})\prod_{j=0}^{k}\{1-\hat{p}(\bar{a}^{user}_{j,v}, \bar{l}_{j,v})\}\{1-\hat{q}(\bar{a}^{user}_{j+1,v}, \bar{l}_{j+1,v})\} \tag{9}$$

In the special case where either the event of interest is not subject to competing events or competing events are treated as censoring events $\hat{q}(\bar{l}_{j+1,v}, \bar{a}^{user}_{j+1,v})$ is set to 0 for all j in (9).

Steps 2 and 3 above can be repeated for multiple user-defined treatment rules h^{user} and risk ratio/risk difference estimates constructed based on a specified reference intervention. The natural course intervention is always automatically implemented and is the default reference unless otherwise specified by the user. Ninety-five percent confidence intervals are computed based on repeating the entire algorithm (Steps 1-3) in B bootstrap samples based on percentiles of the bootstrap distribution for user specified B. The user can also opt to additionally output estimates of the risk (risk ratios/risk differences) by all times $t+1$, $t \leq K$ in addition to the risk by $K+1$.

In addition to the parametric g-formula estimate of the natural course risk, a nonparametric estimate will also be computed automatically and reported in the output. When censoring events are present in the data, this is a completely unadjusted estimate of the natural course risk (i.e. it relies on marginal exchangeability for censoring).[9] When competing risks are absent or treated as censoring events, this is computed as the complement of a product-limit survival estimator[10] in the observed data. When competing risks are present and not treated as censoring events, this is computed using an Aalen Johansen estimator[11]. See Young et al.[2] and Logan et al.[12] for additional details.

Note on coding choice in input data set for Y_{k+1} on line where individual is censored ($C_{k+1} = 1$): Records coded as `NA` will be excluded when fitting the discrete hazard model in step 1.b of the algorithm and in nonparametric estimation of the natural course risk while records coded as 0 will be included in computing these estimates. This choice impacts how "risk sets" at k are defined. It will make no difference to the estimates when the intervals are made small enough such that there are no failures in intervals where there are also censoring events. Otherwise, this choice may impact effect estimates to some degree. Note that by (i) the fact that the algorithms of the previous section do not rely on an estimate of the distribution of censoring and (ii) the required structure of the input data set such that subjects have no more records after they are censored, the input data set does not actually require any variables defining censoring indicators.

B.2 Fixed end of follow-up outcomes

The algorithm for estimating the g-formula for the mean of an outcome at the end of follow-up $K+1$ under a user-specified intervention $h^{user}(\bar{a}_k, a^*_k, \bar{l}_k)$ is nearly identical to that for the risk in the case of survival outcomes described in the previous section but relies on a slightly different input data structure (see Section

2.1.2). In the modified algorithm

Step 1.b. is replaced by

- Modified Step 1.b.: Using only records on line $K+1$, estimate the mean of the outcome at $K+1$ conditional on treatment and covariate history and remaining uncensored to the previous time, $\mu(\bar{l}_K, \bar{a}_K) \equiv \mathsf{E}[Y|\bar{L}_K = \bar{l}_K, \bar{A}_k = \bar{a}_K, \bar{C}_{K+1} = 0]$. This estimate is automatically based on fitting a linear regression model with Y the dependent variable where the user specifies the function of "history" $(\bar{L}_K, \bar{A}_K)$. Because this fit will only use records on line $K+1$ of the data it will not be a function of time. See Section 2.1.7.

Step 2.c. is replaced by:

- Modified Step 2.c.: For $k = K$ only, estimate $\mu(\bar{l}_K, \bar{a}_K)$ based on specifications from step 1.b. Denote this estimate $\hat{\mu}(\bar{l}_{K,v}, \bar{a}^{user}_{K,v})$.

Step 3 is replaced by

- Modified Step 3: Compute the intervention mean estimate as

$$\frac{1}{n}\sum_{v=1}^{n}\hat{\mu}(\bar{a}^{user}_{K,v}, \bar{l}_{K,v}) \tag{10}$$

As discussed above, in this case, if a subject dies prior to $K+1$, this must be treated as a censoring event (D_{k+1} must be considered a component of the vector C_{k+1}, $k = 0, \ldots, K$) and steps 1.c and 2.d are not implemented.

C Additional features

C.1 Specifying pre-baseline lagged values of a time-varying covariate

C.1.1 Required structure of the input dataset

When pre-baseline measures of a time-varying covariates are assumed needed to control for confounding, users can format their data set as follows.

To include a maximum of i pre-baseline times for any particular covariate, users need to add rows for records at times $-i, -i+1, \ldots, -1$ for each subject in addition to the rows for records at follow-up times described in Section 2.1.2. Only the values of the time-varying covariates for which the base history functions (i.e. `lagged`, `cumavg`, and `lagavg`) are applied, individual identifier, and time index need to be specified in rows indexed by these pre-baseline times. Any other data provided in these rows will be ignored in the estimation algorithm.

The example data set `continuous_eofdata_pb` illustrates an appropriately formatted longitudinal data set with pre-baseline times. This data set is of the same format as `continuous_eofdata` (i.e. the data set presented in Example 3) with the addition of 2 pre-baseline records for all subjects. Data for the first subject, who is not censored, is presented below:

```
   id t0 L1         L2 A          Y L3
1:  1 -2  1  0.9796455 1         NA  7
2:  1 -1  2 -1.0441153 1         NA  7
3:  1  0  2 -1.0120653 1         NA  7
4:  1  1  3 -1.0828285 1         NA  7
5:  1  2  2 -0.9985398 1         NA  7
6:  1  3  2 -0.9374480 1         NA  7
```

```
7:  1  4  1 -0.8180795 1        NA  7
8:  1  5  3 -0.8738211 1        NA  7
9:  1  6  1 -0.9414732 0 -1.747542  7
```

When including pre-baseline times, the call to the `gformula` function is not changed. For instance, users can apply the code illustrated in Example 3 using the data set `continuous_eofdata_pb`. The following subsection describes the changes to the algorithm when including pre-baseline times.

C.1.2 Generating covariate histories

When the input data set includes pre-baseline times, the history functions `lagged`, `cumavg`, and `lagavg` are applied as follows.

In the application of the `lagged` function to a covariate named `Zj` to compute its ith lag at time k, recall that `lagi_Zj` is added to the input data set and is set to 0 on lines with $k < i$ when pre-baseline times are not included in the input data set. When pre-baseline times are included, `lagi_Zj` will take the value of Z_j measured at the pre-baseline time, $k - i$.

The `cumavg` function applied to the covariate named `Zj` adds the variable `cumavg_Zj` to the input data set, which contains the cumulative average (from pre-baseline time $-i$) to the current follow-up time $k \geq 0$.

When applying the `lagavg` function to a covariate named `Zj` to compute the ith lag of its cumulative average at time k, the variable `lag_cumavgi_Zj` is added to the input data set and takes on the value of the cumulative average (from pre-baseline time i) to the time $k - i$.

C.2 Specifying covariate distributions, continued

Below, we give a detailed description of each of the pre-programmed options for input to `covtypes` along with required and optional sub-parameters:

- `'binary'`: The mean of the covariate conditional on history (see Section 2.1.5) is estimated via the estimated coefficients of a generalized linear model (GLM) (using `glm` in R), where the family of the GLM is `binomial`. The covariate values are simulated at each time k in step 2 of the estimation algorithm (Appendix B) given the previously simulated history under intervention by sampling from a Bernoulli distribution with parameter this estimated conditional mean. Assuming the covariate in question is the jth entry in `covnames`, then the sub-parameter `covmodels`, which is a vector of length `covnames`, must contain as its jth entry a model statement for estimating the mean of the covariate in the jth entry of `covnames` as a function of history and possibly `time_name`. Optionally, the user can also specify the jth entry of `covlink`, which is a vector containing the link function(s) for the GLM specified in the jth entry of `covmodels`. The default value of `covlink` for the `'binary'` covtype is `logit`. Moreover, users can optionally specify the the jth entry of `control`, which is a vector of lists specifying the parameters of the fitting process of the `glm` function (see `control` argument of the `glm` function). Note that binary covariates in the input data set must be of class numeric and coded as 0 or 1.

- `'normal'`: The mean of the covariate conditional on history is estimated via the estimated coefficients of a GLM where the family is `gaussian`. The covariate values are simulated at each time k in step 2 given the previously simulated history under intervention by sampling from a normal distribution with mean this estimated conditional mean and variance the residual mean squared error from the GLM fit (see Appendix B). Values generated outside the observed range for that covariate over all times k are subsequently set to the minimum or maximum of this range. The sub-parameter `covmodels`, defined as in the `binary` case, must be specified, and the sub-parameters `covlink` and `control` can optionally also be specified. The default value of `covlink` for the `'normal'` covtype is `identity`.

- `'categorical'`: The probability that a covariate with at least 3 levels takes a particular level conditional on history, is estimated via the estimated coefficients of a multinomial logistic regression model.

Specifically, the `multinom` function in the **nnet** package is used to fit the model.[13] In order to fit the model, the covariate must be made a factor in the input data set (e.g., by using the `as.factor` function). The covariate values are simulated at each time k in step 2 given the previously simulated history under intervention by sampling from a multinoulli distribution with parameters these estimated conditional probabilities. The sub-parameter `covmodels` must be specified. For this covariate type, the corresponding entry in `control` is passed into the `nnet` fitting function (rather than the `control` argument of the `glm` function). Similar parameters of the fitting process (e.g., maximum number of iterations `maxit`) can be specified for this covariate type in the `control` argument.

- `'bounded normal'`: The observed covariate values are first standardized to the interval $[0, 1]$, inclusive, by subtracting the minimum value and dividing by the range. Subsequently, the mean of the standardized covariate conditional on history is estimated via the estimated coefficients of a GLM (family `gaussian`). Standardized covariate values are simulated at each time k in step 2 given the previously simulated history under intervention by sampling from a normal distribution with mean this estimated conditional mean and variance the residual mean squared error from the GLM fit. The simulated standardized values are then transformed back to the original scale, and generated values that fall outside the observed range are set to the minimum or maximum of this range. The sub-parameter `covmodels` must be specified. The sub-parameters `covlink` and `control` can optionally be specified. The default value of `covlink` for the `bounded normal` covtype is `identity`.

- `'zero-inflated normal'`: For a covariate with support ≥ 0, the probability that the covariate equals 0 conditional on history is first estimated via the estimated coefficients of a generalized linear model (`glm`) where the family of the GLM is `binomial`. The mean of the covariate values conditional on history and among positive values is then estimated via a GLM where the family is `gaussian`. The simulated covariate values are created by first generating an indicator of whether the covariate value is zero or non-zero from a Bernoulli distribution with the estimated probability of the first model. Covariate values are then generated from a normal distribution with the estimated mean of the second model and multiplied by the generated zero indicator. Non-zero generated covariate values that fall outside the observed range are set to the minimum or maximum of the range *of non-zero observed values* of the covariate. The sub-parameter `covmodels`, defined as before, must be specified, and the sub-parameters `covlink` and `control` can optionally also be specified. The default value of `covlink` for the `'zero-inflated normal'` covtype is `identity`. In this case, the corresponding entry in `control` should be a list of length two, where the first element is a list specifying the parameters of the fitting process of the binomial GLM and the second element is a list specifying the parameters of the fitting process of the Gaussian GLM.

- `'truncated normal'`: The mean of the covariate is estimated via the estimated coefficients of a truncated normal regression model. The `truncreg` function in the **truncreg** package is used to fit the model.[14] The simulated covariate values are generated by sampling from a truncated normal distribution with the estimated covariate mean. Generated values that fall outside the observed range are set to the minimum or maximum of the observed range. The sub-parameter `covmodels`, defined as before, must be specified, as well as the sub-parameter `point`. Assuming the covariate is the jth entry of `covnames`, `point`, which is a vector of length p, must contain as its jth entry the truncation point for the covariate, and the sub-parameter `direction`, which is likewise a vector of length p, must contain as its jth entry the direction(s) of truncation (`'left'` or `'right'`). For this covariate type, the corresponding entry in `control` is passed into the `truncreg.fit` function (rather than the `control` argument of the `glm` function).

- `'absorbing'`: This option is similar to `'binary'`, with the same required and optional sub-parameters. However, in this case, the GLM fit is restricted to records where the value of the covariate at $k-1$ (the lagged value) is 0. Then in the simulation step, once a 1 is first generated, the covariate value at that time and all subsequent times is set to 1. This option applies to a covariate that, once it switches to 1 at time k, it stays 1 at all subsequent times (e.g. an indicator of a disease diagnosis *by* time k.)

- `'categorical time'`: See details for this option in Appendix C.3.

Any sub-parameters required by a specified `covtype` must be specified as a vector element of `covparams` with length equal to the length of `covnames` but some entries in each vector may be `NA`. The following sample syntax illustrates this principle for a dataset with covariates `Z1`, `Z2`, and `Z3`:

```
gformula(..., covnames = c('Z1', 'Z2', 'Z3'),
         basecovs = c('race', 'sex'),
         time_name = 't0',
         histories = c(lagged),
         histvars = list(c('Z1', 'Z2', 'Z3')),
         covtypes = c('categorical', 'truncated normal', 'binary'),
         covparams = list(covmodels = c(Z1 ~ lag1_Z2 + lag1_Z3 + lag1_Z1 +
                                          lag2_Z2 + lag2_Z3 + lag2_Z1 + race +
                                          sex + t0 + I(t0^2),
                                        Z2 ~ Z1 + lag1_Z1 + lag1_Z2 + lag1_Z3 +
                                          lag2_Z2 + lag2_Z3 + race + sex + t0 +
                                          I(t0^2),
                                        Z3 ~ Z1 + Z2+ lag1_Z1 + lag1_Z2 +
                                          lag1_Z3 + lag2_Z3 + race + sex + t0 +
                                          I(t0^2)),
                          covlink = c(NA, NA, 'probit'),
                          point = c(NA, 0.5, NA),
                          direction = c(NA, 'left', NA),
                          control = c(NA, NA, list(maxit = 200))))
```

In this example, all three covariates require a model statement, so the `covmodels` sub-parameter contains three non-`NA` entries. However, only the third covariate, `Z3`, requires the subparameter `covlink` so only the third entry of the `covlink` sub-parameter is non-`NA`. Likewise, only the second covariate, `Z2`, requires the subparameters `point` and `direction` so only the second entries of these subparameters are non-`NA`. Note in this example, by specifying `histories = c(lagged)` and `histvars = c('Z1', 'Z2', 'Z3')`, the variables `lag1_Z1`, `lag1_Z2`, `lag1_Z3`, `lag2_Z1`, `lag2_Z2`, `lag2_Z3`, are automatically created and added to the input data set `obs_data` (see Section 2.1.5) because they are referenced in the model statements in `covmodels`.

Without additional specifications, the distributions of time-varying covariates are fit using records for all times k (i.e. under pooled over time models). Therefore, users will typically want to allow these models to depend on the interval index k (otherwise, the distributions are assumed the same at all k). To allow dependence on time, users can include the time variable `time_name`, or any function of this variable that does not need to be created outside of a model statement in R, in the model statement. For example, in the above sample call, each covariate mean at time k is assumed a quadratic function of k by the inclusion of the terms `t0` and `I(t0^2)`. In Appendix C.3, we describe how to allow these distributions to depend on a function of time that cannot be created within a model statement.

C.3 Allowing distributions to depend on a categorization of time

Users may choose to allow the distributions of time-varying covariates to depend on a categorized function of the time index, e.g. indicators for quartiles of time. To implement this, a new variable based on a categorization of `time_name` must first be created and added to the input data set. The naming convention requires that the categorical time variable has the same name as the continuous time variable, with `_f` appended to the end. For example, the call below creates indicators for quartiles of time, which assumes that the input data set has 8 time points and the time index is called `t0`:

```
thresholds <- c(1, 3, 5)
```

```
basicdata$t0_f <- ifelse(basicdata$t0 <= thresholds[1], 0,
                         ifelse(basicdata$t0 <= thresholds[2], 1,
                                ifelse(basicdata$t0 <= thresholds[3], 2, 3)))
basicdata$t0_f <- as.factor(basicdata$t0_f)
```

While the time index, or any function of this index, is conceptually not a time-varying covariate, when pooled over time models are assumed to be a function of categorized time, the time variable with appendix `_f` must be included as a component of the argument `covnames`. When this is included the corresponding component of `covtypes` should be set to `'categorical time'` and the corresponding component of `covmodels` should be set to `NA`.

Sample syntax is as follows:

```
gformula(..., time_name = 't0',
         covnames = c('L', 'A', 't0_f'),
         histories = c(lagged),
         histvars = list(c('A')),
         covtypes = c('binary', 'binary', 'categorical time'),
         covparams = list(covmodels = c(L ~ lag1_A + lag1_L + t0_f,
                                        A ~ lag1_A + L + lag1_L + t0_f,
                                        NA)))
```

Note that the modified time variable with extension `_f` need only be created and referenced when fewer categories than levels of the original time variable `time_name` are desired by the user. When users choose to define one category per level of `time_name`, an indicator for each level of the time index may be created within the model statement using `as.factor(time_name)` without additional pre-processing steps as illustrated in Section 2.2.

C.4 Bootstrapping and parallelization

The `gformula` function can implement a nonparametric bootstrap to estimate standard errors and 95% confidence intervals. The parameter `nsamples` specifies the number of bootstrap samples. By default, this parameter is set to 0, indicating no bootstrap samples. See Appendix B for details.

Applications of the g-formula can be computationally expensive, especially when a large number of bootstrap samples are generated. To parallelize bootstrapping and estimation under each intervention, set `parallel = TRUE`. When parallelization is used, users must specify the desired number of CPU cores to parallelize across. In many applications, users may wish to set the number of cores equal to the number of total available cores minus one.

The following is sample syntax for applying 500 bootstrap replicates of the g-formula in parallel across the total number of available cores minus one:

```
ncores <- parallel::detectCores() - 1
gformula(..., nsamples = 500, parallel = TRUE, ncores = ncores)
```

C.5 Incorporating deterministic knowledge of the data structure

Sometimes we have information about relationships between time-varying covariates that can be used in place of arbitrary parametric model assumptions or other methods of smoothing to avoid extrapolation where unnecessary. For example, consider the case where we have two time-varying covariates corresponding, respectively, to indicators of whether an individual has started menopause by a given interval k (`menopause`) and whether she is pregnant in interval k (`pregnancy`). In this case, we know that given `menopause == 1`, the probability that `pregnancy == 0` is 1 regardless of other history and can incorporate this knowledge into the algorithm.

Generally, let $Z_{j,k}$ (e.g. pregnancy in interval k) be a component of the covariate vector Z_k for which we have deterministic knowledge of its distribution given its 'history" $(Z_{j-1,k}, \ldots, Z_{1,k}, \overline{L}_{k-1}, \overline{A}_{k-1})$ (e.g. menopause status by interval k defined as a $Z_{h,k}$ where $h < j$). Note this implies a preferred permutation of the components of Z_k, rather than an arbitrary permutation (see Appendix B); specifically, in this example, menopause should precede pregnancy in the chosen permutation. We can incorporate this knowledge as follows into the estimation algorithm described in Appendix B:

- In step 1.a of the algorithm restrict the chosen method of estimating the mean of $Z_{j,k}$ (e.g. pregnancy status) given "history" to only records where deterministic knowledge is absent. In our example, this would be the case for records with `menopause == 0`.
- In step 2.a of the algorithm, set $Z_{j,k}$ deterministically to its known value for histories under which this value is known. Otherwise, draw $Z_{j,k}$ according to the model-based estimate (or otherwise estimated) conditional distribution of $Z_{j,k}$. In our example, if the value of `menopause` in step 2.a. at time k is 1 then `pregnancy` is set to 0. Otherwise, the value of `pregnancy` at time k is drawn from the estimated distribution in step 1.a.

The **gfoRmula** package allows for this type of restricted modeling and modified simulation step using the `restrictions` parameter. To implement the scenario described above, the user will define the following parameters within the the function call:

```
gformula(..., restrictions = list(c('pregnancy', 'menopause == 0',
                                    simple_restriction, 0)))
```

Note that `restrictions` is a list and can contain multiple vectors; that is, the user can impose multiple modeling restrictions.

For each vector in `restrictions`:

- The first entry is the covariate $Z_{j,k}$ (e.g. `pregnancy`) for which we have knowledge of its distribution given a particular "history."
- The second entry is the condition that must be *true* for the conditional mean of the covariate in the first entry to be modeled; equivalently, the condition under which we do *not* have knowledge of the distribution of $Z_{j,k}$ (e.g. `menopause == 0`)
- The third entry is a function that determines the value of the covariate in the first entry when the condition in the second entry is *not* true. In the example above, the `simple_restriction` function simply assigns `pregnancy` a static value when `menopause != 0`.
- The fourth entry is a value used by the function in the third entry. In the above example, this is the value to be assigned to `pregnancy` when `menopause != 0`.
- It is also possible to include additional entries; all of these should be values used by the function in the third entry.

In addition to `simple_restriction`, the package includes an additional carry-forward restriction type named `carry_forward`. Rather than assign a known value, if the condition in the second entry of `restrictions` is not true, `carry_forward` assigns the value of the covariate at the previous time point (that is, it "carries forward" the value from the previous time point if modeling does not occur at the current time point). For example, in some cases certain covariates will only be measured in certain intervals. In this case, if (i) $Z_{j,k}$ is defined as the last measured value of a covariate relative to time k, and (ii) the input data set is constructed so that at times k where the covariate is not measured then $Z_{j,k} = Z_{j,k-1}$ then we have deterministic knowledge of the distribution of $Z_{j,k}$ given $Z_{j,k-1}$ at those times. For example, suppose a covariate `smoking` corresponds to the last measured value of smoking status and is only measured in intervals $k = 0$, 3, and 6. To incorporate the fact that the last measured value of smoking status at $k \neq 0, 3, 6$ must be equal to the value at $k - 1$ at those times, we incorporate the following syntax:

```
gformula(..., time_name = 't0',
         restrictions = list(c('smoking', 't0%in%c(0, 3, 6)',
                                carry_forward)))
```

Note that this approach, of course, requires that exchangeability assumptions for the causal effect of interest hold given only last measured values of certain covariates (see Appendix A.3).

Simple restrictions may also be placed on the outcome hazard/mean (and the competing event hazard, allowed for survival outcomes only). `yrestrictions`, the parameter for specifying restrictions on the outcome mean/hazard, restricts the estimation for the outcome probability in step 1 to records meeting a specified condition. Then in step 2, if the condition is met based on the previously simulated data, a model-based estimate of the conditional hazard/mean is used. Otherwise, if the condition is not met, the mean/hazard is set to a specified value. (`compevent_restrictions` operates analogously for the competing event.) Restrictions on the outcome or competing event may be used in conjunction with covariate restrictions.

As an example, suppose the user is interested in estimating the risk of ventilator-associated pneumonia by 30-day follow-up. The criteria for this outcome includes being on the ventilator for at least 3 days. Suppose the user is interested in effects on risk of ventilator-associated pneuomonia in a population of ventilated patients of treatment interventions beginning on the first day of ventilation, with k indexing days. In this case, the event of interest Y_{k+1} cannot take the value 1 until $k = 3$ such that the hazard in the first two intervals of follow-up is 0 by definition. To incorporate this knowledge, the function call would include:

```
gformula(..., time_name = 'time', yrestrictions = list(c('time>2', 0)))
```

As with `restrictions`, the `yrestrictions` and `compevent_restrictions` parameters each accept a list of vectors, meaning that it is possible to impose multiple restrictions on the simulation of each variable by including multiple vectors within each list.

C.6 Attaching a visit process to a covariate

In clinical cohorts, the data are not usually recorded at regular intervals but rather are recorded every time the patient visits the clinic. Therefore, the times at which the time-varying covariates are measured will vary by subject. In this setting, it is typical to construct the data such that at a time when there is no visit/measurement, the last measured value of a covariate is carried forward (such that $Z_{j,k}$ actually corresponds to the last measured value of a covariate). Furthermore, the user may choose to censor a subject if he or she is not seen after a specified consecutive number of time intervals. Following Hernán et al.[1], under such measurement processes, the visit process history through k generally should itself be considered part of the confounder history ($\overline{L}_k$).

Following Young et al.[9], in these settings where (i) the visit/measurement process is itself defined as a time-varying confounder (i.e., is conceptually a component of L_k); (ii) the input data set is constructed such that last measured values are carried forward at times of no new measurements; and (iii) the user constructs the input data set such that a subject is censored after a certain number of consecutive times with no visit/measurement, there are certain deterministic relationships in the data that can be incorporated into both step 1 and step 2 of the parametric g-formula estimation algorithm to potentially reduce model misspecification and increase precision. First, by (ii), we know that if there is no visit/measurement in interval k then $Z_{j,k} = Z_{j,k-1}$ (i.e., $Z_{j,k}$ is always equal to its lagged value). We also know that, by (iii), for s the max number of consecutive missed visits since the last visit allowed before a subject is censored, the indicator of a visit at time k (itself a component of L_k), by (ii) must be 1 if the number of consecutive missed visits since the last visit at $k - 1$ is s. The determinisms described in (ii) and (iii) above can in principle be incorporated into the algorithm via the parameters described in Appendices C.5 and C.8.

Alternatively, the deterministic knowledge in (ii) and (iii) can be incorporated via the parameter `visitprocess`, a list parameter taking vector entries. The first element of each vector gives the name of a time-varying indicator in the input data set of whether a covariate was measured in interval k. The second entry gives the

name of that covariate. The conditional mean of the covariate given the "history" in step 1 will be estimated only for records where there is a current visit. If the visit indicator equals 1, then in step 2, the value of the dependent covariate will be generated from a distribution based on this estimate; otherwise, the last value is carried forward. The third parameter is the max number s of missed measurements of this covariate allowed since the last measurement before a subject is censored. The probability of a visit given the history in step 1 of the algorithm is estimated only using records where the sum of consecutive missed visits through $k-1$ is less than s. In step 2, if the sum of consecutive missed visits through $k-1$ is less than s, then the visit indicator is simulated from a distribution based on this estimate; otherwise, the visit indicator is set to 1 so as to eliminate subjects with more than s consecutive missed visits.

In the following example, a time-varying covariate in the input data set, `visit`, indicates whether CD4 cell count and viral load lab measurements were taken in interval k, and `cd4` and `rna` are the respective covariates for CD4 cell count and viral load. The input data set is constructed such that a subject is censored after 3 consecutive missed lab measurements (i.e. `visit == 0` 3 intervals in a row). In this case the call will include:

```
gformula(..., covnames = c('visit', 'cd4', 'rna'),
         covtypes = c('binary', 'normal', 'normal'),
         visitprocess = list(c('visit', 'cd4', 3),
                             c('visit', 'rna', 3)))
```

Note that `visit` itself must be included in `covnames` and assigned a value in `covtypes`.

The above example assumes that CD4 cell count and viral load are either both measured or both not measured at each visit. The following call alternatively allows a data set with separate visit process indicators for CD4 cell count and viral load and separate maximum missed visits determining censoring.

```
gformula(..., covnames = c('visit_cd4', 'cd4', 'visit_rna', 'rna'),
         covtypes = c('binary', 'normal', 'binary', 'normal'),
         visitprocess = list(c('visit_cd4', 'cd4', 4),
                             c('visit_rna', 'rna', 5)))
```

Note in the current version (i.e., version 0.3.0) of the package, a covariate attached to a visit process must be measured (i.e. the indicator of measurement must be 1) at baseline for all individuals in the data set.

C.7 Specifying a custom `covtype`

In addition to the existing `covtypes`, users are also able to choose their own covariate distributions and methods of estimating their parameters by setting the `covtype` equal to `'other'` and supplying their own fit and prediction functions through the parameters `covfits_custom` and `covpredict_custom`, respectively.

A user-written fit function must take the parameters `covparams` (see Section 2.1.6), `covname` (the name of the covariate), `obs_data` (the name of the input data set), and `j` (the index of the covariate). The template for a custom fit function is:

```
fit_custom <- function(covparams, covname, obs_data, j){
  covmodels <- covparams$covmodels
  otherparam1 <- covparams$otherparam1
  otherparam2 <- covparams$otherparam2
  fit <- fitfunc(formula = as.formula(paste(covmodels[j])), a = otherparam1[j],
                 b = otherparam2[j])
  return (fit)
}
```

A user-written prediction function must take the parameters `obs_data` (the observed data), `newdf` (the simulated dataset at time `t`), `fitcov` (the model object for the covariate), `time_name` (the name of the time variable), `t` (the current time index), `condition` (any condition restricting the modeling of the covariate),

`covname` (the name of the covariate), and any number of additional parameters, which are specified in the original `gformula` function call. An example format is given below. Suppose the inputs to `...` are `a = 1`, `b = 0.5`:

```
predict_custom <- function(obs_data, newdf, fitcov, time_name, t, condition,
                           covname, ...){
  n <- dim(newdf)[1]
  theta <- predict(fitcov, type = 'response', newdata = newdf)
  extra_args <- list(...)
  a <- extra_args$a
  b <- extra_args$b
  prediction <- predictfunc(n = n, theta = theta, a = a, b = b)
  return (prediction)
}
```

Like all sub-parameters corresponding to the covariates, `covfits_custom` and `covpredict_custom` are vectors of the same length and in the same order as `covnames`. At the index where the custom `covtype` is desired, `covfits_custom` and `covpredict_custom` will contain the names of the user-supplied fit and prediction functions. Multiple custom covtypes can be specified as well.

For example, suppose the user wishes to model a covariate using random forests. Then their respective fit and predict function might look like:

```
fit_rf <- function(covparams, covname, obs_data, j){
  covmodels <- covparams$covmodels
  importance <- covparams$importance
  maxnodes <- covparams$maxnodes
  ntree <- covparams$ntree
  fit <- randomForest::randomForest(formula = as.formula(paste(covmodels[j])),
                                    data = obs_data, importance = importance[j],
                                    maxnodes = maxnodes[j], ntree = ntree[j])
  return (fit)
}

predict_rf <- function(obs_data, newdf, fitcov, time_name, t, condition,
                       covname, ...){
  extra_args <- list(...)
  proximity <- extra_args$proximity
  prediction <- predict(object = fitcov, newdata = newdf,
                        proximity = proximity)
  return (prediction)
}
```

Here, `fit_rf` requires the sub-parameters `importance`, `maxnodes`, and `ntree`. Likewise, `predict_rf` requires the parameter `proximity`. Therefore, the call to the gformula should follow the format:

```
gformula(..., covnames = c('L1', 'L2', 'A'),
         covtypes = c('binary', 'custom', 'zero-inflated normal'),
         covparams = list(covmodels = c(L1 ~ lag1_L1 + lag1_L2 + lag1_A,
                                        L2 ~ L1 + lag1_L1 +lag1_L2 + lag1_A,
                                        A ~ L1 + lag1_L1 + L2 + lag1_L2 +
                                          lag1_A),
                          covlink = c('logit', NA, 'identity'),
```

```
                        importance = c(NA, TRUE, NA),
                        maxnodes = c(NA, 4, NA),
                        ntree = c(NA, 30, NA)),
         covfits_custom = c(NA, fit_rf, NA),
         covpredict_custom = c(NA, predict_rf, NA),
         proximity = FALSE)
```

To use custom covariate fit and predict functions in conjunction with the `parallel` option, users should take care to load any packages used by those custom functions into the global environment using `library(<packagename>)` prior to calling the `gformula` function, as the packages will not be successfully exported to the clusters otherwise.

C.8 Specifying a custom history

If users wish to generate histories other than the three provided by the package, they may write those functions themselves. These functions take parameters `pool` (a `data.table` containing the simulated data up until the current time point `t`), `histvars` (the name of the time-varying covariates for which functions of histories will be created), `time_name` (the name of the time variable), `t` (the current time index), and `id_name` (the name of the ID variable). A history function generates in `pool` the value of the desired function of history of each covariate listed in `histvars` at time `t` based on the covariates values at times prior to (and, possibly, at time) `t`. The function does not return a new dataframe; rather, it modifies `pool` in place to reduce memory use. No object should be returned by a custom history function.

An example function that generates the average of the three most recent values (i.e., the current value and the last two lagged values for $t > 1$, the current value and the last lag for $t = 1$ and the current value for $t = 0$) is given below:

```
ave_last3 <- function(pool, histvars, time_name, t, id_name){
  i <- min(c(t, 2))

  # Get indicators for individuals in the (observed or simulated) data set at time t
  current_ids <- unique(pool[get(time_name) == t][[id_name]])

  # At time t, for each element (histvar) in histvars, create / update
  # the column called ave_last3_<histvar> which contains the average of
  # the three most recent values of histvar
  lapply(histvars, FUN = function(histvar){
    pool[get(time_name) == t,
         (paste("ave_last3_", histvar, sep = "")) :=
           as.double(tapply(pool[get(id_name) %in% current_ids &
                                  get(time_name) <= t &
                                  get(time_name) >= t-i][[histvar]],
                            pool[get(id_name) %in% current_ids &
                                  get(time_name) <= t &
                                  get(time_name) >= t-i][[id_name]],
                            FUN = sum) / (i + 1))]
  })
}
```

In general, users will have to create an indicator for individuals in the data set at time t, as illustrated in the above example. This is because the custom history function must work not only on the simulated data, which contains $K + 1$ records for every value of `id`, but also on observed data, which may include fewer records per `id`. In this case, custom history functions will typically use such an indicator to obtain the past covariate values for the individuals in the data set at time t.

To use this newly written function in a `gformula` call, the user may write, for example:

```
gformula(..., covnames = c('L1', 'L2', 'A'),
         covparams = list(covmodels = c(L1 ~ lag1_L1 + lag1_L2 + lag1_A + t0,
                                        L2 ~ L1 + lag1_L1 + lag1_L2 + lag1_A +
                                          t0,
                                        A ~ ave_last3_L1 + ave_last3_L2 +
                                          lag1_A + t0)),
         histories = c(lagged, ave_last3),
         histvars = list(c('L1', 'L2', 'A'), c('L1', 'L2')))
```

C.9 Specifying a custom intervention

Beyond the two interventions provided, `static` and `threshold`, users may also write their own intervention functions. These must accept the parameters `newdf` (a `data.table` containing the simulated dataset at time `t`), `pool` (a `data.table` containing the simulated dataset at times prior to `t`, i.e. from `0, ..., t - 1`), `intvar` (the name of the time-varying covariate to be intervened on), `intvals` (a list of one or more values needed internally by the intervention function), `time_name` (the name of the time variable), and `t` (the current time index).

Several technical considerations should be made when writing custom interventions functions.

- As with custom history functions, no objects should be returned by custom intervention functions. Instead, custom intervention functions should modify `newdf` by reference (i.e., using the `:=` operator from `data.table`). The following examples illustrate how to add and update columns by reference for a custom intervention.
- `newdf` is initiated to the simulated data set at time $t-1$. Prior to calling the custom intervention function, the values of the time-varying covariates are updated to their simulated values at time t. Consequently, any auxiliary columns added to `newdf` (e.g., columns `cond_met_ever` and `cond_tracker` in the example `dyn_int` below) are initially set to their values at time $t-1$. Users should therefore only modify auxiliary columns if their values need to be updated from time $t-1$. Moreover, the value of the treatment covariate at time t is initially set to its simulated value under the natural course intervention. Therefore, users should not update the value of the treatment covariate in cases where the natural course is desired.

We now give two examples of user-defined intervention functions that implement dynamic interventions; interventions that depend on time-updated values of covariates:

C.9.1 Example 1: a dynamic intervention based on the current value of a time-varying covariate

The following is an example of a function that defines an intervention under which treatment is given only for individuals where `L2` is above a certain threshold (provided in `intvals`):

```
example_intervention <- function(newdf, pool, intvar, intvals, time_name, t){
  newdf[(intvar) := 0]
  newdf[L2 < intvals[[1]], (intvar) := 1]
}
```

Supposing the user wants to set the threshold to 0.75, the function call would then be:

```
gformula(..., intvars = list('A'),
         interventions = list(list(c(example_intervention, 0.75))))
```

C.9.2 Example 2: a (random) dynamic intervention based on a function of the history of a time-varying covariate

The following is an example of a function that defines a more complex dynamic intervention "start combined antiretroviral therapy (cART) within m months if CD4 cell count first drops below x" for some grace period of $m > 0$ months (assuming intervals k are one month long) and for some cut off x.[9,15] During the grace period, treatment is assigned according to the observed distribution of treatment given an individual's past measured treatment and confounder history. In the data set, the column `lncd4` corresponds to the natural logarithm of the CD4 cell count and the column `art` corresponds to an indicator of cART initiation (`art=1` indicates treatment initiation).

```
dyn_int <- function(newdf, pool, intvar, intvals, time_name, t){
  threshold <- intvals[[1]]
  m <- intvals[[2]]

  # Determine whether threshold has ever been met and track when it first occurs
  if (t == 0){
    newdf[lncd4 < threshold, `:=` (cond_met_ever = 1, cond_tracker = t)]
    newdf[lncd4 >= threshold, cond_met_ever := 0]
  } else {
     newdf[cond_met_ever == 0 & lncd4 < threshold, `:=`
     (cond_met_ever = 1, cond_tracker = t)]
  }
  # If treatment has been initiated by time t-1, then it has been initiated by time t
  if (t > 0){
    newdf[pool[get(time_name) == (t - 1), get(intvar) == 1], (intvar) := 1]
  }

  # If threshold has never been met, set treatment to 0.
  newdf[cond_met_ever == 0, (intvar) := 0]

  # If threshold was met at time t-m, set treatment at t to 1
  if (t >= m){
    newdf[cond_tracker <= (t - m), (intvar) := 1]
  }
}
```

To set a threshold of 350 for CD4 cell count and a grace period of $m = 6$ months, the function call would be:

```
gformula(..., intvars = list('art'),
         interventions = list(list(c(dyn_int, log(350), 6))))
```

To verify that the custom intervention function is working properly, users should inspect the simulated data set under the intervention. For instance, below are simulated data for the first ten time points for two "individuals" (or, more precisely, simulated histories) under this random dynamic treatment regime:

```
    month art    lncd4 cond_met_ever cond_tracker
 1:     0   0 6.142037             0           NA
 2:     1   0 5.973712             0           NA
 3:     2   0 5.885895             0           NA
 4:     3   0 5.694269             1            3
 5:     4   1 5.732024             1            3
```

```
 6:     5   1 5.620208             1            3
 7:     6   1 5.485906             1            3
 8:     7   1 5.666780             1            3
 9:     8   1 5.479236             1            3
10:     9   1 5.514820             1            3
```

The simulated data above is consistent with the desired intervention rule because the condition to start treatment is met at time (`month`) 3 and treatment (`art`) is started at time 4 which is within the grace period (6 months) of meeting the condition to start.

```
    month art    lncd4 cond_met_ever cond_tracker
 1:     0   0 5.669881             1            0
 2:     1   0 5.874165             1            0
 3:     2   0 5.603337             1            0
 4:     3   0 5.699042             1            0
 5:     4   0 5.850501             1            0
 6:     5   0 5.775751             1            0
 7:     6   1 5.719197             1            0
 8:     7   1 5.765443             1            0
 9:     8   1 5.695890             1            0
10:     9   1 5.859916             1            0
```

The simulated data above is consistent with the desired intervention rule because the condition to start treatment is met at time 0 and treatment is started at time 6, the end of the grace period.

C.10 Hazard ratios

For survival outcomes only, users have the options of calculating the hazard ratio comparing two interventions using the argument `intcomp`. If, for example, the user wishes to calculate the hazard ratio between the natural course and the second intervention specified in the parameter `interventions`, then `intcomp` should be set as `intcomp = list(0, 2)`. When bootstrapping is used (i.e., by setting `nsamples > 0`), a 95% confidence interval for the hazard ratio is then also computed. Note that the hazard ratio calculation is available only for survival analysis. When competing events are present in the data and the parameters `compevent_model` and `compevent_name` are specified, the *subdistribution* hazard ratio[16] is computed. Otherwise, if these are not specified, a quantity that, depending on underlying assumptions, can be interpreted as either a *marginal* or *cause-specific* hazard ratio is computed[2,17]. We include these hazard ratios as options for users but generally discourage reporting these as causal effect measures because even counterfactual contrasts in any of these versions of the hazard ratio generally do not have a causal interpretation.[2,18,19]

C.11 Summary of key arguments to the `gformula` function

- `id`: Character string specifying the name of the ID variable in `obs_data`.
- `time_points`: Number of time points to simulate. By default, this argument is set equal to the maximum number of records that `obs_data` contains for any individual.
- `obs_data`: Data table containing the observed data.
- `seed`: Starting seed for simulations and bootstrapping.
- `nsimul`: Number of subjects for whom to simulate data. By default, this argument is set equal to the number of subjects in `obs_data`.
- `time_name`: Character string specifying the name of the time variable in `obs_data`.

- outcome_name: Character string specifying the name of the outcome variable in obs_data.
- compevent_name: Character string specifying the name of the competing event variable in obs_data. Only applicable for survival outcomes.
- intvars: List, whose elements are vectors of character strings. The kth vector in intvars specifies the name(s) of the variable(s) to be intervened on in each round of the simulation under the kth intervention in interventions.
- interventions: List, whose elements are lists of vectors. Each list in interventions specifies a unique intervention on the relevant variable(s) in intvars. Each vector contains a function implementing a particular intervention on a single variable, optionally followed by one or more "intervention values" (i.e., integers used to specify the treatment regime).
- int_descript: Vector of character strings, each describing an intervention. It must be in same order as the entries in interventions.
- ref_int: Integer denoting the intervention to be used as the reference for calculating the risk ratio and risk difference. 0 denotes the natural course, while subsequent integers denote user-specified interventions in the order that they are named in interventions. The default is 0.
- covnames: Vector of character strings specifying the names of the time-varying covariates in obs_data.
- covtypes: Vector of character strings specifying the "type" of each time-varying covariate included in covnames. The possible "types" are: "binary", "normal", "categorical", "bounded normal", "zero-inflated normal", "truncated normal", "absorbing", "categorical time", and "custom".
- covparams: List of vectors, where each vector contains information for one parameter used in the modeling of the time-varying covariates (e.g., model statement, family, link function, etc.). Each vector must be the same length as covnames and in the same order. If a parameter is not required for a certain covariate, it should be set to NA at that index.
- covfits_custom: Vector containing custom fit functions for time-varying covariates that do not fall within the pre-defined covariate types. It should be in the same order covnames. If a custom fit function is not required for a particular covariate (e.g., if the first covariate is of type "binary" but the second is of type "custom"), then that index should be set to NA. The default is NA.
- covpredict_custom: Vector containing custom prediction functions for time-varying covariates that do not fall within the pre-defined covariate types. It should be in the same order as covnames. If a custom prediction function is not required for a particular covariate, then that index should be set to NA. The default is NA.
- histvars: List of vectors. The kth vector specifies the names of the variables for which the kth history function in histories is to be applied.
- histories: Vector of history functions to apply to the variables specified in histvars. The default is NA.
- ymodel: Model statement for the outcome variable.
- yrestrictions: List of vectors. Each vector containins as its first entry a condition and its second entry an integer. When the condition is TRUE, the outcome variable is simulated according to the fitted model; when the condition is FALSE, the outcome variable takes on the value in the second entry. The default is NA.

- `compevent_restrictions`: List of vectors. Each vector containins as its first entry a condition and its second entry an integer. When the condition is `TRUE`, the competing event variable is simulated according to the fitted model; when the condition is `FALSE`, the competing event variable takes on the value in the second entry. The default is `NA`. Only applicable for survival outcomes.

- `restrictions`: List of vectors. Each vector contains as its first entry a covariate for which *a priori* knowledge of its distribution is available; its second entry a condition under which no knowledge of its distribution is available and that must be `TRUE` for the distribution of that covariate given that condition to be estimated via a parametric model or other fitting procedure; its third entry a function for estimating the distribution of that covariate given the condition in the second entry is false such that *a priori* knowledge of the covariate distribution is available; and its fourth entry a value used by the function in the third entry. The default is `NA`.

- `visitprocess`: List of vectors. Each vector contains as its first entry the covariate name of a visit process; its second entry the name of a covariate whose modeling depends on the visit process; and its third entry the maximum number of consecutive visits that can be missed before an individual is censored. The default is `NA`.

- `compevent_model`: Model statement for the competing event variable. The default is `NA`. Only applicable for survival outcomes.

- `intcomp`: List of two numbers indicating a pair of interventions to be compared by a hazard ratio. The default is `NA`, resulting in no hazard ratio calculation.

- `sim_data_b`: Logical scalar indicating whether to return the simulated data set. If bootstrap samples are used (i.e., `nsamples` is set to a value greater than 0), this argument must be set to `FALSE`. The default is `FALSE`.

- `nsamples`: Integer specifying the number of bootstrap samples to generate. The default is 0.

- `parallel`: Logical scalar indicating whether to parallelize simulations of different interventions to multiple cores.

- `ncores`: Integer specifying the number of CPU cores to use in parallel simulation. This argument is required when parallel is set to `TRUE`. In many applications, users may wish to set this argument equal to `parallel::detectCores() - 1`.

D Benchmark against the GFORMULA SAS macro

We benchmarked the **gfoRmula** R package with the **GFORMULA** SAS macro when applied to simulated data based on the HIV Causal Collaboration data[20]. This data set consists of longitudinal data of 5,000 individuals followed for up to 60 months. We applied the parametric g-formula to estimate the risk of death under the interventions of “Never treat with antiretroviral therapy” and “Always treat with antiretroviral therapy”. The data set and code used to apply the g-formula for this comparison are available at https://github.com/CausalInference/gfoRmula-benchmark.

We compared the estimated 5-year risks of death between the R package and SAS macro. The estimated 5-year risk of death was comparable between the two implementations; the R package estimated a 5-year risk of death of 0.0888 under the “Never treat” intervention and 0.0452 under the “Always treat” intervention, whereas the SAS macro estimated a 5-year risk of death of 0.0878 under the “Never treat” intervention and 0.0444 under the “Always treat” intervention.

We evaluated the computation time of the **gfoRmula** R package and **GFORMULA** SAS macro in two settings: (1) no bootstrapping, and (2) 10 bootstrap samples. We applied the **gfoRmula** R package and the SAS macro without parallelization or multithreading to make a fair comparison. In both settings, the

programs were applied on a Dell R730 PowerEdge Server with 2x8 core XEON E5-2667 3.2 GHz hyper-threaded CPUs, 320 GB of memory, and approximately 50 TB of disk space that runs Centos 7.7 X86_64, R version 3.4.3, and SAS version 9.4. The R implementation took 3.01 minutes when no bootstrap samples were used and took 27.71 minutes when 10 bootstrap samples were used. The SAS implementation took 0.62 minutes when no bootstrap samples were used and took 4.95 minutes when 10 bootstrap samples were used.

References

[1] Hernán, M. A., McAdams, M., McGrath, N., Lanoy, E., & Costagliola, D. (2009). Observation plans in longitudinal studies with time-varying treatments. Statistical Methods in Medical Research, 18, 27–52.

[2] Young, J., Stensrud, M., Tchetgen Tchetgen, E., & Hernán, M. (2020). A causal framework for classical statistical estimands in failure time settings with competing events [Online publication date: 27 January 2020]. Statistics in Medicine. https://doi.org/10.1002/sim.8471

[3] Young, J. G., Hernán, M. A., & Robins, J. M. (2014). Identification, estimation and approximation of risk under interventions that depend on the natural value of treatment using observational data. Epidemiologic Methods, 3, 1–19.

[4] Young, J. G., Logan, R. W., Robins, J. M., & Hernán, M. A. (2019). Inverse probability weighted estimation of risk under representative interventions in observational studies. Journal of the American Statistical Association, 114, 938–947.

[5] Richardson, T. S., & Robins, J. M. (2013). Single World Intervention Graphs (SWIGs): A unification of the counterfactual and graphical approaches to causality. Center for Statistics and the Social Sciences, University of Washington Series.

[6] Hernán, M. A., & Robins, J. (2020). Causal Inference: What If. (Chapman & Hall/CRC).

[7] Robins, J. M. (1986). A new approach to causal inference in mortality studies with a sustained exposure period: Application to the healthy worker survivor effect [Errata (1987) in Computers and Mathematics with Applications 14, 917-921. Addendum (1987) in Computers and Mathematics with Applications 14, 923-945. Errata (1987) to addendum in Computers and Mathematics with Applications 18, 477.]. Mathematical Modelling, 7, 1393–1512.

[8] Robins, J. M., Hernán, M. A., & Siebert, U. (2004). Effects of multiple interventions, In M. Ezzati, A.D. Lopez, A. Rodgers, C.J.L. Murray (eds.), Comparative Quantification of Health Risks: Global and Regional Burden of Disease Attributable to Selected Major Risk Factors. (World Health Organization).

[9] Young, J. G., Cain, L. E., Robins, J. M., O'Reilly, E. J., & Hernán, M. A. (2011). Comparative effectiveness of dynamic treatment regimes: An application of the parametric g-formula. Statistics in Biosciences, 3, 119–143.

[10] Kaplan, E., & Meier, P. (1958). Nonparametric estimation from incomplete observations. Journal of the American Statistical Association, 53, 457–81.

[11] Aalen, O., & Johansen, S. (1978). An Empirical Transition Matrix for Non-Homogeneous Markov Chains Based on Censored Observations. Scandinavian Journal of Statistics, 5, 141–150.

[12] Logan, R., Young, J., Taubman, S., Lodi, S., Picciotto, S., Danaei, G., & Hernán, M. A. (2016). GFORMULA SAS MACRO. https://www.hsph.harvard.edu/causal/software

[13] Venables, W. N., & Ripley, B. D. (2002). Modern applied statistics with s (Fourth). (Springer).

[14] Croissant, Y., & Zeileis, A. (2018). truncreg: Truncated gaussian regression models. R package version 0.2-5. https://CRAN.R-project.org/package=truncreg

[15] Cain, L. E., Robins, J. M., Lanoy, E., Logan, R., Costagliola, D., & Hernán, M. A. (2010). When to start treatment? a systematic approach to the comparison of dynamic regimes using observational data. International Journal of Biostatistics, 6.

[16] Fine, J., & Gray, R. (1999). A proportional hazards model for the subdistribution of a competing risk. Journal of the American Statistical Association, 94, 496–509.

[17] Kalbfleisch, J. D., & Prentice, R. L. (1980). The Statistical Analysis of Failure Time Data. (John Wiley).

[18] Hernán, M. A., Hernández-Diáz, S., & Robins, J. M. (2004). A structural approach to selection bias. Epidemiology, 15, 615–625.
[19] Hernán, M. A. (2010). The hazards of hazard ratios. Epidemiology, 21, 13–15.
[20] Ray, M., Logan, R., Sterne, J. A., Hernández-Díaz, S., Robins, J. M., Sabin, C., Bansi, L., vanSighem, A., de Wolf, F., Costagliola, D., ..., and Hernán, M. A. on behalf of The HIV-CAUSAL Collaboration. (2010). The effect of combined antiretroviral therapy on the overall mortality of HIV-infected individuals. AIDS, 24, 123–37.
